# Spatial and temporal control over photoresponsive nanoclusters

**DOI:** 10.1093/nsr/nwag053

**Published:** 2026-01-28

**Authors:** Ying Xu, Mengfan Chang, Hao Li, Ning Zhang, Siqi Li, Pu Wang, Yong Pei, Xi Kang, Manzhou Zhu

**Affiliations:** Department of Chemistry and Centre for Atomic Engineering of Advanced Materials, Key Laboratory of Structure and Functional Regulation of Hybrid Materials of Ministry of Education, Anhui Province Key Laboratory of Chemistry for Inorganic/Organic Hybrid Functionalized Materials, Anhui University, Hefei 230601, China; Department of Chemistry and Centre for Atomic Engineering of Advanced Materials, Key Laboratory of Structure and Functional Regulation of Hybrid Materials of Ministry of Education, Anhui Province Key Laboratory of Chemistry for Inorganic/Organic Hybrid Functionalized Materials, Anhui University, Hefei 230601, China; School of Materials and Chemical Engineering, Anhui Jianzhu University, Hefei 230601, China; State Key Laboratory of Opto-Electronic Information Acquisition and Protection Technology, Anhui University, Hefei 230601, China; State Key Laboratory of Opto-Electronic Information Acquisition and Protection Technology, Anhui University, Hefei 230601, China; Department of Chemistry, Key Laboratory of Environmentally Friendly Chemistry and Applications of Ministry of Education, Xiangtan University, Xiangtan 411105, China; Department of Chemistry, Key Laboratory of Environmentally Friendly Chemistry and Applications of Ministry of Education, Xiangtan University, Xiangtan 411105, China; Department of Chemistry and Centre for Atomic Engineering of Advanced Materials, Key Laboratory of Structure and Functional Regulation of Hybrid Materials of Ministry of Education, Anhui Province Key Laboratory of Chemistry for Inorganic/Organic Hybrid Functionalized Materials, Anhui University, Hefei 230601, China; Department of Chemistry and Centre for Atomic Engineering of Advanced Materials, Key Laboratory of Structure and Functional Regulation of Hybrid Materials of Ministry of Education, Anhui Province Key Laboratory of Chemistry for Inorganic/Organic Hybrid Functionalized Materials, Anhui University, Hefei 230601, China

**Keywords:** atomically precise nanoclusters, photoinduced conversion, solid-state transformation, alloying effect, spatial control, temporal control

## Abstract

Although cluster species undergo efficient photoresponsive transformations in dilute solutions, their solid-state materials suffer from severely impeded responsiveness due to insufficient motional freedom. Here, we present a photochemical approach that enables spatial and temporal control over nanocluster structure/size conversions in the crystalline state. The Cu_18_ nanocluster, whether in solution or in solid form, undergoes a photoinduced transformation when exposed to 365-nm light, resulting in a size-reduced Cu_14_ nanocluster. The single-atom alloy counterpart, Ag_1_Cu_17_, possesses a remarkably enhanced efficiency towards the photoinduced conversion to form the same cluster product. The comparable photoinduced conversion efficiencies between Cu_18_ and Ag_1_Cu_17_ are monitored by using time-dependent characterizations and further rationalized by using theoretical calculations. The high photoconversion efficiency of crystalline nanocluster materials allows the precise spatial and temporal control of solid-state transformations at the micrometer scale by using femtosecond cold laser technology or by controlling the irradiation time of ultraviolet light. This study introduces a novel pair of clusters with comparable photoinduced conversion characteristics, allowing atomic-level characterizations and an in-depth understanding of the photochemical behavior of metal nanoclusters. Furthermore, the findings in this work are expected to facilitate the design of cluster-based solid-state nanomaterials for downstream photoresponsive applications.

## Teaser

A novel structure-correlated Cu-based nanocluster pair with comparable photoinduced conversion efficiencies was presented, allowing for spatial control over solid-state transformations.

## INTRODUCTION

Photoresponsive materials exhibit light-controllable optical, electrical, chemical, mechanical and morphological properties, demonstrating potential applications in chemical sensing, encryption, information storage and optoelectronics [1–5]. The realization of these unique functionalities critically depends on the precise integration of specific photoresponsive molecules within the material. For example, the dimerization and dissociation of coumarin, the ring-opening and ring-closing isomerization of spiropyran and diarylethene, as well as the *cis*–*trans* isomerization of azobenzene constitute canonical photoresponsive behaviors that have been widely investigated [6–10]. However, the witching behavior of photoresponsive materials is intrinsically state-dependent: while species in dilute solutions undergo efficient light-induced structural changes due to sufficient motional freedom, their solid-state counterparts suffer from severely impeded responsiveness. This arises from the restricted molecular mobility and fixed spatial orientations imposed by tight packing, which can suppress or even preclude photo transformations [2]. Therefore, overcoming the limitations imposed by the solid-state environment to design novel materials capable of efficient and rapid photoresponse within confined spaces remains challenging.

Metal nanoclusters, as condensed matter that lies between molecules and nanocrystals, exhibit discrete electronic energy levels and ultrahigh reactivity [11–21]. In contrast to traditional photo-switching materials that rely on single-molecule isomerization, atomically precise metal nanoclusters exhibit a multilevel photoresponse mechanism in which photoinduced transformations arise from the synergistic effects of ligand isomerization, electronic structure modulation within the metal core and structural reorganization of the cluster framework. This well-defined, atomic-level structure–property relationship enables cluster-based nanomaterials to serve as a unique platform for advanced intelligent photoresponsive applications [22–26]. It is noteworthy that excellent research progress has been reported on photoinduced structural conversion, especially in silver-cluster-based solution systems due to their prominent dynamic equilibrium characteristic when exposed to light [27–31]. Less is known about the photoresponsive behavior of solid-state nanoclusters, probably resulting from their intrinsically restricted molecular motions and weak optical transmittance. Furthermore, previously reported cluster cases involving photoinduced conversion have primarily focused on the conversion path between cluster precursors and products, while the conversion efficiency has been rarely considered. The development of structure-correlated cluster pairs that display comparable capabilities and previously reported cluster cases involving photoinduced conversion have primarily focused on the conversion path between cluster precursors and products, while the conversion efficiency has been rarely considered. The development of structure-correlated cluster pairs that display comparable capabilities and efficiencies towards photoinduced conversion is of paramount importance if an in-depth understanding of such solid-state photoirradiation-induced size/structure transformations is to be realized.

**Scheme 1.**
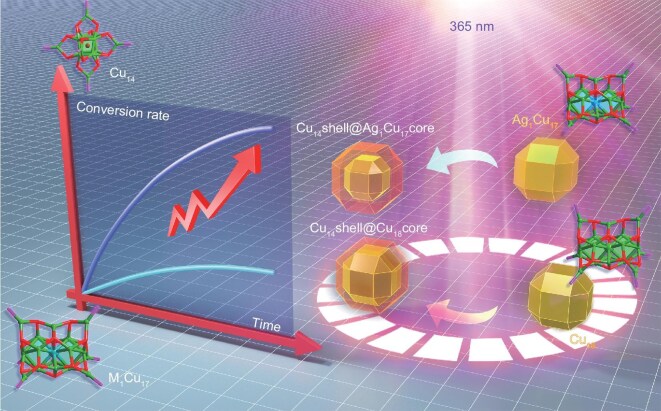
A novel structure-correlated Cu-based nanocluster pair with comparable photoinduced conversion efficiencies is presented, allowing spatial control over solid-state transformations.

Herein, we achieve the atomic-level regulation of photoinduced conversion efficiency from both spatial and temporal aspects in cluster-based solid-state materials M_1_Cu_17_H_2_(SPh*^p^*F)_15_[P(Ph*^p^*F)_3_]_6_(SbF_6_)_1_ (M = Cu/Ag; Cu_18_ or Ag_1_Cu_17_ for short) through single-atom alloying (Scheme 1). Under 365-nm photoexcitation, the M_1_Cu_17_ nanoclusters undergo a size-reduction transformation to {Cl_1_Cu_14_(SPh*^p^*F)_12_[P(Ph*^p^*F)_3_]_6_}^+^ (Cu_14_ for short), derived from optical absorption and mass spectrometry analyses. The single-atom-alloyed Ag_1_Cu_17_ nanocluster displays a 3-fold conversion efficiency relative to its homolog Cu_18_ under the same conditions, which is further rationalized by using theoretical calculations. Besides, M_1_Cu_17_ functions as an excellent photosensitive single-crystal platform, which can undergo phase transition from both spatial and temporal aspects under light irradiation. The controllable photoinduced structural transformation of these cluster-based solid-state materials would open new channels for downstream photoresponsive applications.

## RESULTS AND DISCUSSION

### Synthesis and structural details

**Figure 1.**
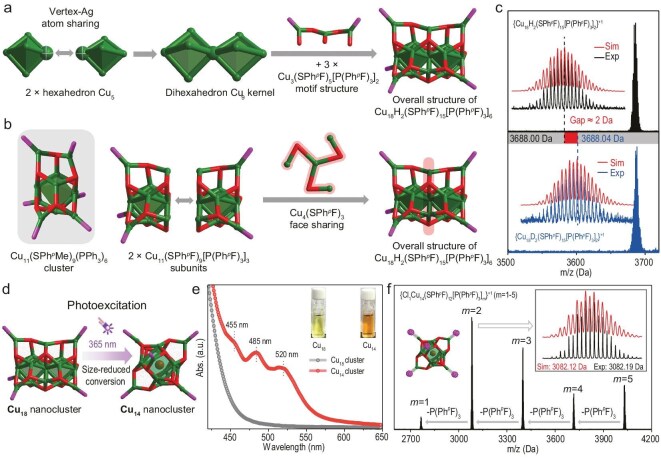
Structural anatomy and mass characterization of Cu_18_ and Cu_14_ nanoclusters. (a) Total structure of Cu_18_ contained two hexahedron Cu_5_ cores by sharing a vertex Cu atom and the obtained dihexahedron Cu_9_ kernel was further stabilized by three Cu_1_(SPh*^p^*F)_5_[P(Ph*^p^*F)_3_]_2_ motif structures. (b) Crystal structure of the Cu_11_(SPh*^p^*Me)_9_(PPh_3_)_6_ nanocluster and the overall structure of Cu_18_, which could be viewed as the assembly of two Cu_11_(SPh*^p^*F)_9_[P(Ph*^p^*F)_3_]_3_ subunits by sharing a Cu_4_(SPh*^p^*F)_3_ face. (c) ESI-MS results of Cu_18_ (black line) and Cu_18_-D (blue line) and their calculated isotope patterns (red lines). (d) Scheme illustration of the transformation from Cu_18_ to Cu_14_ under 365-nm photoexcitation. (e) Comparison of the optical absorptions between the Cu_18_ and Cu_14_ nanoclusters. Insets: photos of the CH_2_Cl_2_ solutions of nanoclusters. (f) ESI-MS results of the Cu_14_ nanocluster. Insets: experimental mass result of {Cl_1_Cu_14_(SPh*^p^*F)_12_[P(Ph*^p^*F)_3_]_2_}^+1^ (black line) and its calculated isotope pattern (red line); scheme illustration of the easy-to-dissociate state of phosphine ligands on the Cu_14_ cluster surface. Color labels: green, Cu; magenta, P; red, S; brown, Cl. For clarity, all C, F and H atoms are omitted.

The Cu_18_ nanocluster is prepared by directly reducing Cu–SPh*^p^*F–P(Ph*^p^*F)_3_ complexes with NaBH_4_ (see Scheme S1A). Structurally, the Cu_18_ nanocluster contains a dihexahedral Cu_9_ kernel that is stabilized by three Cu_3_(SPh*^p^*F)_5_[P(Ph*^p^*F)_3_]_2_ staple-like motif structures (Fig. 1a). Alternatively, the overall structure of the Cu_18_ nanocluster can be viewed as the assembly of two Cu_11_(SPh*^p^*F)_9_[P(Ph*^p^*F)_3_]_3_ subunits sharing a Cu_4_(SPh*^p^*F)_3_ face (Fig. 1b). A Cu_11_(SPh*^p^*Me)_9_(PPh_3_)_6_ nanocluster with an analogous configuration to the Cu_11_(SPh*^p^*F)_9_[P(Ph*^p^*F)_3_]_3_ subunit has been reported by ignoring their different thiol and phosphine ligands (Fig. 1b) [32]. The structural comparison between the Cu_11_(SPh*^p^*F)_9_[P(Ph*^p^*F)_3_]_3_ subunit in Cu_18_ and Cu_11_(SPh*^p^*Me)_9_(PPh_3_)_6_ is performed. Although the bond lengths between the two Cu_11_ structures are similar, the corresponding bond angles vary remarkably (Fig. S1). The bond angles in the terminal Cu_1_(SR)_2_(PR’_3_)_1_ unit (R’=Ph or Ph_p_F) are more uneven in the Cu_11_(SPh*^p^*F)_9_[P(Ph*^p^*F)_3_]_3_ subunit in Cu_18_, illustrating its more twisted configuration relative to Cu_11_(SPh*^p^*Me)_9_(PPh_3_)_6_, probably due to the removal of three phosphine ligands in the former structure.

The composition of the Cu_18_ nanocluster is then analysed via electrospray ionization mass spectrometry (ESI-MS). The mass result of Cu_18_ shows an intense signal at 3686.00 Da in the positive mode, matching well with the chemical formula of Cu_18_H_2_(SPh*^p^*F)_15_[P(Ph*^p^*F)_3_]_2_ with a +1-charge state (Fig. 1c and Fig. S2). Of note, the detected formulas of the Cu_18_ nanocluster are determined as {Cu_18_H_2_(SPh*^p^*F)_15_[P(Ph*^p^*F)_3_]_2_]*_n_*}^+1^, with *n* ranging from 1 to 3, demonstrating a dissociating characteristic of P(Ph*^p^*F)_3_ ligands on the cluster surface (Fig. S2), which has also been observed in other phosphine-stabilized metal nanoclusters [33–35]. Besides, two hydride ligands exist in the Cu_18_ structure. For verifying the presence of two hydrides in Cu_18_, NaBD_4_ is used to realize the deuteration of the Cu_18_ nanocluster, giving rise to the Cu_18_D_2_(SPh*^p^*F)_15_[P(Ph*^p^*F)_3_]_6_ nanocluster (Cu_18_-D for short; Scheme S1B). The mass peak of Cu_18_-D is located at 3688.04 Da, corresponding to {Cu_18_D_2_(SPh*^p^*F)_15_[P(Ph*^p^*F)_3_]_2_}^+1^ (Fig. 1c and Fig. S3). The 2-Da increase when comparing the mass signal of Cu_18_-D with that of Cu_18_ further demonstrates the presence of two hydride ligands in the nanocluster. The presence of two hydride ligands is further verified by using nuclear magnetic resonance (NMR) spectroscopy (Fig. S4) and their locations are determined by using density functional theory calculations (Fig. S5).

The Cu_18_ could maintain its cluster framework under general conditions (i.e. exposed to the atmosphere). Interestingly, under 365-nm photoexcitation, the photoinduced cluster conversion occurs to produce a size-reduced Cu_14_ nanocluster (Fig. 1d). Despite several attempts, the quality of the obtained single crystal is still inadequate for single-crystal diffraction analysis. Fortunately, the optical absorption characterization and the dissociation mass patterns observed through ESI-MS show a strong consistency with those reported for Cu_14_ [34]. The overall structure of the Cu_14_ nanocluster is supposed to be consistent with the reported one, including a semi-hexahedral Cl_1_Cu_8_ kernel that was stabilized by six Cu_1_(SPh*^p^*F)_2_[P(Ph*^p^*F)_3_]_1_ motif-like structures from each face (Fig. S6) [36,37]. These motif-like structures were also widespread in Cu_18_ and the same coordination modes (μ_1_-P and μ_3_-S) facilitated the generation of photoproducts. The innermost Cl of Cu_14_ might have originated from the CH_2_Cl_2_ solvent (Fig. S7), which has been reported in other halogen-protected metal nanoclusters [41–43]. The ultraviolet-visible spectroscopy (UV–vis) results of the Cu_18_ and Cu_14_ nanoclusters are compared. As shown in Fig. 1e, no optical absorption is observed for the CH_2_Cl_2_ solution of Cu_18_; by comparison, three apparent UV–vis signals occur for Cu_14_ at 455, 485 and 520 nm. At the same time, the solution color alters from the pale yellow of Cu_18_ to the orange of Cu_14_. In addition, ESI-MS is performed on Cu_14_ to verify its molecular structure. The five detected mass signals, located at 2766.11, 3082.19, 3400.26, 3716.33 and 4032.40 Da, match well with the chemical formula of {Cl_1_Cu_14_(SPh*^p^*F)_12_[P(Ph*^p^*F)_3_]*_m_*}^+1^, where *m* ranged from 1 to 5 (Fig. 1f).

The quantum-sized effect of the metal nanoclusters renders them prominent nanomaterials with controllable physicochemical properties [11–21]. In this context, any perturbations in the compositions of the clusters may induce tremendous variations in their properties. Accordingly, we perceive a good opportunity to construct a structure-correlated nanocluster series with comparable capabilities and efficiencies towards photoinduced conversion. The Ag-alloyed Ag_1_Cu_17_ nanocluster is controllably prepared by doping the homo-copper Cu_18_ with Ag–P(Ph*^p^*F)_3_ complexes or *in situ* reducing the Ag–Cu–SPh*^p^*F– P(Ph*^p^*F)_3_ complexes with NaBH_4_ (see Scheme S1C and D). Structurally, the introduced Ag heteroatom is located at the innermost position of the cluster framework and the shared Cu_4_(SPh*^p^*F)_3_ face in Cu_18_ is substituted by Ag_1_Cu_3_(SPh*^p^*F)_3_ (Fig. S8). By comparing the geometric structures of Cu_18_ and Ag_1_Cu_17_, we found that the incorporation of the innermost Ag atoms expanded the core–shell configurations by extending the corresponding M–Cu or M–Ag bones (Fig. S9), probably resulting from the larger atomic size of Ag than Cu. The X-ray photoelectron spectroscopy results of Ag_1_Cu_17_ identify the successful introduction of the Ag heteroatom into the nanocluster (Fig. S10). ESI-MS results of Ag_1_Cu_17_ and its deuterated Ag_1_Cu_17_D_2_(SPh*^p^*F)_15_[P(Ph*^p^*F)_3_]_6_ further evidence the presence of two hydride ligands in the Cu_18_ cluster framework (Figs S11–S13). Besides, in addition to the single-Ag-doped Ag_1_Cu_17_, mass signals of undoped Cu_18_ and multi-Ag-doped Ag*_x_*Cu_18__–_*_x_* (*x* = 2–4) are detected, demonstrating the flexibility of this cluster framework.

### Photoinduced structural conversion and temporal control

Under 365-nm photoexcitation, the Ag_1_Cu_17_ could also convert to the size-reduced Cu_14_ nanocluster, much like the Cu_18_ nanocluster. The *in situ* tracking of the transformation from Ag_1_Cu_17_ to Cu_14_ is performed and monitored by using ESI-MS and ^2^H NMR. For a CH_2_Cl_2_ solution of Ag_1_Cu_17_, four tracking samples are collected with a 30-second interval under photoexcitation. As shown in Fig. S14, only the mass signals of Ag_1_Cu_17_ are detected at the beginning of the photoinduced cluster conversion (Stage 1). With the process of photoexcitation, the peaks of Ag_1_Cu_17_ disappear gradually and, in the meantime, the mass signals of Cu_14_ emerge (Stages 2 and 3). Finally, all Ag_1_Cu_17_ cluster precursors transform to the Cu_14_ nanocluster (Stage 4). In addition, time-dependent ^2^H NMR spectra also validate the gradual conversion from Ag_1_Cu_17_ to Cu_14_ (Fig. S15). Because the hydride ligands only exist in Ag_1_Cu_17_ while Cu_14_ contained no hydrides, the hydride signals of Ag_1_Cu_17_ at 5.00 and 5.11 ppm weaken gradually. At the same time, an intense ^2^H NMR peak at 3.81 ppm emerged, which might have originated from the hydride-containing metal complexes. Indeed, some metal-hydride-based fragments should be produced with the photoinduced size reduction from Ag_1_Cu_17_ to Cu_14_. Furthermore, control experiments confirmed that this transformation is a direct photochemical process, as it proceeds independently of ambient oxygen or temperature (Figs S16 and S17). These results collectively demonstrate that the structure conversion is driven by photoexcitation.

**Figure 2.**
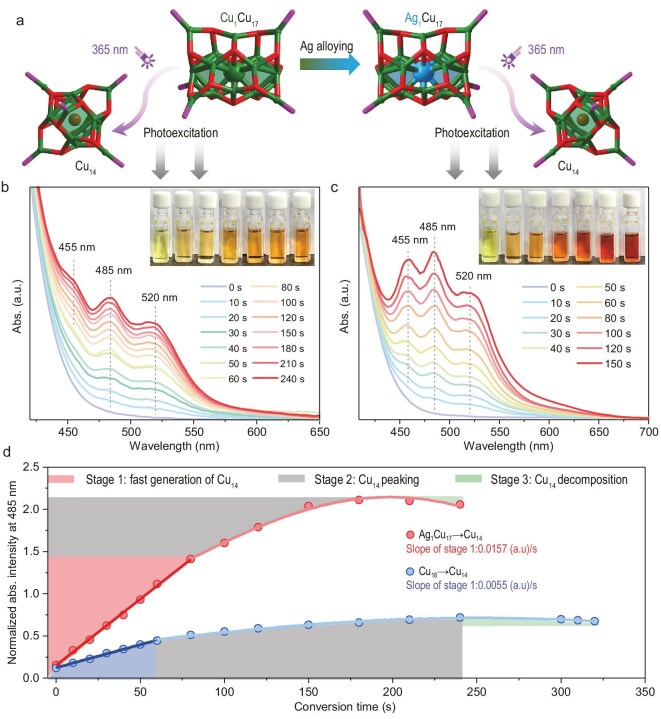
Comparison of the photoinduced conversion from Cu_18_ or Ag_1_Cu_17_ to Cu_14_. (a) Scheme illustration of the nanocluster conversion under 365-nm photoexcitation. Color labels: green, Cu; blue, Ag; magenta, P; red, S; brown, Cl. For clarity, all C, F and H atoms are omitted. (b) Time-dependent optical absorption of the photoinduced conversion from Cu_18_ to Cu_14_. (c) Time-dependent optical absorption of the photoinduced conversion from Ag_1_Cu_17_ to Cu_14_. Insets: photos of the time-variant nanocluster solutions. For the experiment, the initial concentration of Cu_18_ and Ag_1_Cu_17_ was 2.5 mg/mL. (d) Time-dependent concentration of the photoinduced-generated Cu_14_ nanoclusters from Cu_18_ or Ag_1_Cu_17_.

In this context, a M_1_Cu_17_ cluster pair with a homologous structure framework is established and their efficiencies in the photoinduced conversion to the Cu_14_ nanocluster are investigated (Fig. 2a). Under the same photoexcitation conditions, the time-dependent UV–vis spectra of the two conversions are monitored. For the photoinduced conversion from Cu_18_ to Cu_14_, the characteristic absorption peaks at 455, 485 and 520 nm grow gradually within 240 seconds and the solution color alters to orange over time (Fig. 2b). By comparison, the Ag_1_Cu_17_ displays a faster conversion to Cu_14_ under 365-nm photoexcitation and it only takes 40 seconds to achieve the same intensity of absorption peaks as that of the Cu_18_-based conversion (Fig. 2c). In addition, a more obvious UV–vis spectrum of Cu_14_ appeared as the reaction continued, demonstrating a stronger capability of the photoinduced conversion of Ag_1_Cu_17_ relative to Cu_18_. Furthermore, the solution of Ag_1_Cu_17_ undergoes an immense color change along with the photoinduction, i.e. from pale yellow to dark red, further manifesting the high photoinduced conversion efficiency of the Ag-doped Ag_1_Cu_17_ nanocluster.

For further differentiating the photoinduced conversion efficiency between the Ag_1_Cu_17_ and Cu_18_ nanoclusters, we monitored the optical absorption intensity at 485 nm to characterize the generation of the Cu_14_ nanocluster by considering the evident enhancement of the UV–vis absorption at such a point from Ag_1_Cu_17_ or Cu_18_ (with almost no absorption) to Cu_14_ (with strong absorption). Indeed, the intensity of the optical absorptions of metal nanoclusters should be directly proportional to their concentration in solutions, which is in agreement with the Beer–Lambert law [38,39]. As shown in Fig. 2d, for both photoinduced conversion cases, the Cu_14_ nanoclusters are generated rapidly at the beginning (Stage 1) and the conversion rate gradually decreases over time (Stage 2). Eventually, the concentration of Cu_14_ obtained from Ag_1_Cu_17_ is almost four times that of Cu_18_. In addition, the absorptions at 485 nm slightly weaken with continued photoexcitation (Stage 3), suggesting the metastable state of Cu_14_. Indeed, Cu_14_ would be decomposed gradually in solution with the extension of time (Fig. S18). The higher photoconversion rate of Ag_1_Cu_17_ reflects its unique kinetic advantage: the silver-doped framework promotes the faster formation of the metastable intermediate Cu_14_, allowing it to accumulate to a greater extent before the competitive degradation pathway becomes dominant. However, this limited stability significantly constrains its practical applications in solution-phase systems. In addition, we found that the photoinduced generations of Cu_14_ in Stage 1 followed first-order equations and the initial rates (slopes) of the two cases were compared―the conversion rate from Ag_1_Cu_17_ to Cu_14_ was almost three times that of the Cu_18_-based conversion. Consequently, the photoinduced conversion efficiency from the Cu_18_ nanocluster to Cu_14_ has been remarkably improved by substituting its innermost Cu kernel with Ag. In addition, we conducted additional replicate experiments to confirm the experimental reproducibility of the photoconversion process (Fig. S19). For a better understanding of the comparable photoinduced conversion efficiency between Cu_18_ and Ag_1_Cu_17_, theoretical efforts have been made to rationalize the Ag-doped-induced efficiency enhancement (Figs S20 and S21). The theoretical calculations demonstrate that, although Ag doping would not significantly change the electronic structure of Cu_18_, it markedly enhances the structural instability in the excited state. The theoretical calculations demonstrate that, although Ag doping would not significantly change the electronic structure of Cu_18_, it markedly enhances the structural instability in the excited state (S_1_). As shown in Fig. S21A and B, the first singlet excited-state structures of both clusters showed significant deformation compared with their ground-state (S_0_) structures. A detailed analysis of the bond-length variations between the internal H_2_M_1_Cu_11_S_3_ (M = Cu or Ag) kernel and the external Cu_17_S_12_P_6_ shell atoms was performed to evaluate the deformation in the S_1_ structures. The numbers and distances of the broken H–M, Cu–S and Cu–M (M = Cu or Ag) bonds in the S_1_ structure of Ag_1_Cu_17_ were significantly higher than those of Cu_18_ (Fig. S21C–E), suggesting greater deformation in the H_2_Ag_1_Cu_11_S_3_ core of the Ag_1_Cu_17_ cluster. In this context, under photoinduced conditions, the S_1_ structure of Ag_1_Cu_17_ was less stable and more prone to convert to the Cu_14_ cluster.

### Solid-state transformation and spatial control

**Figure 3.**
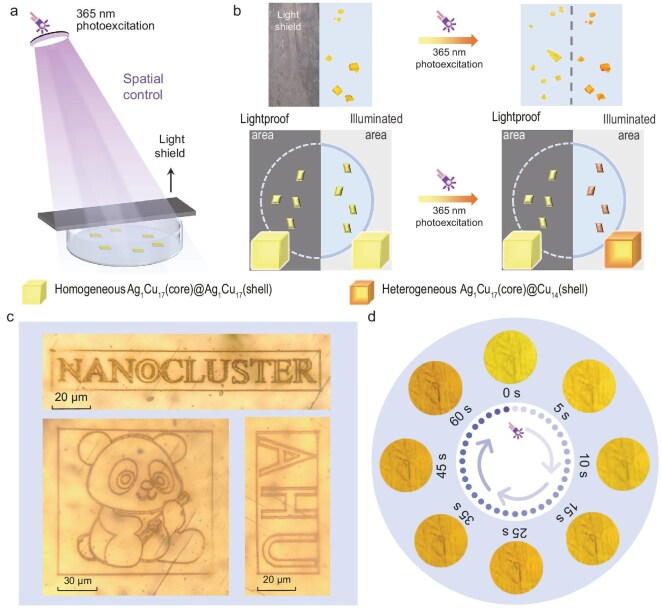
Spatial and temporal control over photoinduced cluster conversion from Ag_1_Cu_17_ to Cu_14_. (a) Device diagram of the spatial control. (b) Color changes of the nanocluster crystal before and after 365-nm photoexcitation in lightproof or illuminated areas. Insets: illustrations of simulated cluster crystals with homogeneous or heterogeneous configurations. (c) Photographs of micrometer-scale patterns microfabricated on the surface of an Ag_1_Cu_17_ crystal using a femtosecond laser. (d) Time-dependent photographs of photoinduced color evolution in Ag_1_Cu_17_ crystal upon UV irradiation.

Compared with solution syntheses with molecular inducers, photoinduced synthesis has the potential to accomplish solid-phase preparation. Besides, the photochemical approach enables spatial control over cluster-structure/size conversions [40]. To attain these goals, we set up a spatial control experiment by arranging some crystals of Ag_1_Cu_17_ on a glass sheet with partially lightproof areas (Fig. 3a). Under 365-nm photoexcitation, only the crystals in the selected illuminated areas of the glass sheet underwent a color change from yellow to orange, demonstrating the occurrence of solid-state cluster transformation. By comparison, the crystals in the lightproof areas maintained their color as yellow (Fig. 3b). To further demonstrate spatial control over the photoinduced conversion of Ag_1_Cu_17_ cluster crystals, we selected elongated single crystals with regular morphology as observation objects. With the assistance of the light shield and the thickness of the crystal itself, we successfully achieved selective color change in approximately one-quarter of the crystal, showing significant spatially resolved photoresponse characteristics (Fig. S22). In addition, Ag_1_Cu_17_ crystalline powder-based thin films (10 wt%, loaded in polymethyl methacrylate) also show excellent responsiveness to ultraviolet light (Figs S23 and S24). Furthermore, precise spatial control at the micro-/nanometer scale was achieved by using femtosecond cold laser technology. As illustrated in Fig. 3c, letters and fine patterns with feature sizes of <150 μm were successfully inscribed on the crystal surface. Scanning electron microscope images further revealed that these laser-written structures possess nanoscale dimensions (Fig. S25), with statistical analysis showing an average line width of 400 nm and a minimum feature size of 310 nm (Fig. S26)—both significantly below the theoretical diffraction limit of the optical system. These results demonstrate the outstanding suitability of Ag_1_Cu_17_ as a solid-state photoconversion material for high‑precision micro‑/nanofabrication.

Compared with the solution state, the response of crystalline M_1_Cu_17_ to ultraviolet light is somewhat diminished. In this context, restricted intermolecular distances and fixed molecular orientations increase the difficulty of realizing photoinduced phase transitions. Fortunately, the cooperative effect of the Ag atom is applicable not only in dilute solutions, but also in the crystalline state, highlighting the unique advantages of alloying. Consistently with photoresponsive results in the solution state, the photo-responsiveness of Ag_1_Cu_17_ is found to be significantly superior to that of the homo-copper analogous Cu_18_. Therefore, Ag_1_Cu_17_ crystals were chosen as a model system for studying the time-dependent behavior of photoinduced phase transitions. By precisely controlling the irradiation time of the ultraviolet light and employing a digital camera to record the evolution of the crystal color over time (Fig. 3d and Fig. S27), slight color changes were observed within 5 seconds, indicating temporal photoresponsivity. However, the extent of the response remained limited by the intrinsic spatial constraints of the solid material, resulting in relatively weak final discoloration. In addition, the limitation is more pronounced in homo-copper Cu_18_ nanoclusters (Fig. S28).

To further verify the solid-state transformation of the crystals with changed colors and figuring out whether the same photoinduced conversion occurred as that in solutions (i.e. from Ag_1_Cu_17_ to Cu_14_), UV–vis spectra and the ESI-MS results of such color-changed and color-maintained crystals (dissolved in CH_2_Cl_2_) were detected. Both the characteristic optical absorptions (455, 485 and 520 nm; as shown in Fig. S29A) and the isotopic mass peaks (3082.19, 3400.26, 3716.33 and 4032.40 Da; as shown in Fig. S29B) of the color-changed samples validate the solid-state conversion from Ag_1_Cu_17_ to Cu_14_. By comparison, the UV–vis and ESI-MS results of the color-maintained crystal samples are still sourced from Ag_1_Cu_17_ completely. Nevertheless, the less obvious optical absorptions and mixed mass signals of the color-changed crystal samples indicate their hybrid compositions and incomplete photoinduced conversions, which is reasonable given that it was difficult for the excitation light to penetrate the cluster crystals to their interior. Besides, the generated Cu_14_ in the outer layers could prevent the Ag_1_Cu_17_ clusters in the inner layers from being stimulated by the excitation light. In this context, the color-changed crystals should follow a heterogeneous Ag_1_Cu_17_ (core)@Cu_14_ (shell) configuration, while that for the color-maintained crystals is homogeneous. The time-dependent fluorescence and micro-Raman spectroscopy data support this inference, as the spectral changes clearly indicate structural heterogeneity (Figs S30 and S31). Accordingly, the crystallographic diffraction of the color-changed crystals was unsuccessful due to their heterogeneous and long-range non-ordered composition.

## CONCLUSIONS

In summary, spatial and temporal control over photoresponsive nanoclusters has been accomplished. Single-atom alloying was exploited to finely regulate the photoinduced conversion efficiency of nanoclusters, observed not only in dilute solutions, but also in the crystalline state. These phenomena were monitored by using time-dependent UV–vis, ESI-MS and NMR spectroscopy and further rationalized by using theoretical calculations, allowing atomic-level characterizations and a deeper understanding of the photoinduced conversion process. Additionally, the spatial and temporal control of the solid-state transformations of such nanoclusters was implemented by exciting cluster crystals under controlled conditions of lightproof/illuminated and irradiation duration. Overall, this study presented a structural-correlated nanocluster pair that enables the elucidation of correlations between cluster structures and photoinduced conversion efficiency at the atomic level. Besides, the findings of this research may provide valuable insights for the fabrication of cluster-based solid-state nanomaterials with programmable compositions and desirable photoresponsive properties.

## Supplementary Material

nwag053_Supplemental_Files
