# Supplementary material for: Spatial and temporal control over photoresponsive nanoclusters
Source: Natl Sci Rev. 2026 Jan 28;13(5):nwag053. doi: 10.1093/nsr/nwag053 (PMC12912717; doi:10.1093/nsr/nwag053)

checkCIF/PLATON report

You have not supplied any structure factors. As a result the full set of tests cannot be run.

THIS REPORT IS FOR GUIDANCE ONLY. IF USED AS PART OF A REVIEW PROCEDURE FOR PUBLICATION, IT SHOULD NOT REPLACE THE EXPERTISE OF AN EXPERIENCED CRYSTALLOGRAPHIC REFEREE.

No syntax errors found. CIF dictionary Interpreting this report

Datablock: 1

```
Bond precision:   C-C = 0.0236 A          Wavelength=1.54186

Cell:            a=32.2725(12)    b=36.3369(11)     c=36.3503(13)
                 alpha=90         beta=94.148(3)    gamma=90
Temperature:     120 K
```

	Calculated	Reported
Volume	42516(3)	42516(3)
Space group	I 2/a	I 1 2/a 1
Hall group	-I 2ya	-I 2ya
Moiety formula	C198 H134 Ag Cu17 F33 P6 S15, F6 Sb [+ solvent]	C198 H134 Ag Cu17 F33 P6 S15, 2(F3 Sb0.5)
Sum formula	C198 H134 Ag Cu17 F39 P6 S15 Sb [+ solvent]	C198 H134 Ag Cu17 F39 P6 S15 Sb
Mr	5230.71	5230.56
Dx,g cm-3	1.634	1.634
Z	8	8
Mu (mm-1)	5.999	5.999
F000	20752.0	20752.0
F000'	20589.29	
h,k,lmax	37,41,41	37,41,41
Nref	33917	33536
Tmin,Tmax	0.399,0.517	0.341,0.518
Tmin'	0.295	

```
Correction method= # Reported T Limits: Tmin=0.341 Tmax=0.518
AbsCorr = MULTI-SCAN

Data completeness= 0.989          Theta(max)= 62.500
```

R(reflections)= 0.1197(17740)   wR2(reflections)= 0.3881(33536)

S = 1.257   Npar= 2310

The following ALERTS were generated. Each ALERT has the format
test-name_ALERT_alert-type_alert-level.
Click on the hyperlinks for more details of the test.

Alert level A

```
PLAT434_ALERT_2_A Short Inter HL..HL Contact F066     ..F066     .       2.00 Ang.
                                          1/2-x,y,1-z  =      2_556 Check
```

Author Response: F066 is the same atom, and the distance between adjacent molecules formed by symmetrical stacking is very close, so the distance between F066 and F066 is correspondingly small.

Alert level B

```
PLAT084_ALERT_3_B High wR2 Value (i.e. > 0.25) ..................       0.39 Report
```

Author Response: The crystal quality is poor, and it is difficult to cultivate crystal with better quality.

```
PLAT342_ALERT_3_B Low Bond Precision on  C-C Bonds ..............    0.02358 Ang.
```

Alert level C

```
THETM01_ALERT_3_C  The value of sine(theta_max)/wavelength is less than 0.590
            Calculated sin(theta_max)/wavelength =    0.5753
PLAT082_ALERT_2_C High R1 Value .................................       0.12 Report
PLAT213_ALERT_2_C Atom Cu0K             has ADP max/min Ratio .....        3.4 prolat
PLAT213_ALERT_2_C Atom S013             has ADP max/min Ratio .....        3.3 prolat
PLAT220_ALERT_2_C NonSolvent   Resd 1  C   Ueq(max)/Ueq(min) Range        3.2 Ratio
PLAT230_ALERT_2_C Hirshfeld Test Diff for    S00W     --C046     .        6.3 s.u.
PLAT230_ALERT_2_C Hirshfeld Test Diff for    S012     --C04T     .        6.6 s.u.
PLAT230_ALERT_2_C Hirshfeld Test Diff for    S013     --C05L     .        6.4 s.u.
PLAT230_ALERT_2_C Hirshfeld Test Diff for    P011     --C05D     .        6.0 s.u.
PLAT234_ALERT_4_C Large Hirshfeld Difference Cu0K     --P016     .       0.17 Ang.
PLAT234_ALERT_4_C Large Hirshfeld Difference S00V     --C02U     .       0.16 Ang.
PLAT234_ALERT_4_C Large Hirshfeld Difference S010     --C02A     .       0.16 Ang.
PLAT234_ALERT_4_C Large Hirshfeld Difference P015     --C050     .       0.17 Ang.
PLAT234_ALERT_4_C Large Hirshfeld Difference P015     --C07C     .       0.16 Ang.
PLAT234_ALERT_4_C Large Hirshfeld Difference F01C     --C03A     .       0.16 Ang.
PLAT234_ALERT_4_C Large Hirshfeld Difference F01W     --C05P     .       0.16 Ang.
PLAT234_ALERT_4_C Large Hirshfeld Difference F02V     --C03N     .       0.25 Ang.
PLAT234_ALERT_4_C Large Hirshfeld Difference F039     --C04S     .       0.18 Ang.
PLAT241_ALERT_2_C High   'MainMol' Ueq as Compared to Neighbors of       Cu0K Check
PLAT241_ALERT_2_C High   'MainMol' Ueq as Compared to Neighbors of       S013 Check
PLAT242_ALERT_2_C Low    'MainMol' Ueq as Compared to Neighbors of       P016 Check
```

```
PLAT242_ALERT_2_C Low    'MainMol' Ueq as Compared to Neighbors of       C05W Check
PLAT242_ALERT_2_C Low    'MainMol' Ueq as Compared to Neighbors of       C05C Check
PLAT242_ALERT_2_C Low    'MainMol' Ueq as Compared to Neighbors of       C065 Check
PLAT242_ALERT_2_C Low    'MainMol' Ueq as Compared to Neighbors of       C05O Check
PLAT242_ALERT_2_C Low    'MainMol' Ueq as Compared to Neighbors of       C048 Check
PLAT242_ALERT_2_C Low    'MainMol' Ueq as Compared to Neighbors of       C04S Check
PLAT242_ALERT_2_C Low    'MainMol' Ueq as Compared to Neighbors of       C070 Check
PLAT242_ALERT_2_C Low    'MainMol' Ueq as Compared to Neighbors of       C07U Check
PLAT242_ALERT_2_C Low    'MainMol' Ueq as Compared to Neighbors of       C03N Check
PLAT242_ALERT_2_C Low    'MainMol' Ueq as Compared to Neighbors of       C157 Check
PLAT242_ALERT_2_C Low    'MainMol' Ueq as Compared to Neighbors of       C06B Check
PLAT242_ALERT_2_C Low    'MainMol' Ueq as Compared to Neighbors of       C07B Check
PLAT242_ALERT_2_C Low    'MainMol' Ueq as Compared to Neighbors of       C07Q Check
PLAT244_ALERT_4_C Low    'Solvent' Ueq as Compared to Neighbors of       Sb02 Check
PLAT260_ALERT_2_C Large Average Ueq of Residue Including      Sb0E      0.165 Check
PLAT334_ALERT_2_C Small <C-C> Benzene Dist.   C01Q     -C03K     .       1.37 Ang.
PLAT434_ALERT_2_C Short Inter HL..HL Contact F033     ..F055     .       2.56 Ang.
                                        1-x,1/2+y,1/2-z  =      4_555 Check
```

Author Response: F066 is the same atom, and the distance between adjacent molecules formed by symmetrical stacking is very close, so the distance between F066 and F066 is correspondingly small.

Alert level G

```
PLAT002_ALERT_2_G Number of Distance or Angle Restraints on AtSite          2 Note
PLAT003_ALERT_2_G Number of Uiso or U(i,j)  Restrained non-H-Atoms        244 Report
PLAT042_ALERT_1_G Calc. and Reported MoietyFormula Strings  Differ     Please Check
              Calc: C198 H134 Ag Cu17 F33 P6 S15, F6 Sb
              Rep.: C198 H134 Ag Cu17 F33 P6 S15, 2(F3 Sb0.5)
PLAT072_ALERT_2_G SHELXL First  Parameter in WGHT  Unusually Large       0.20 Report
PLAT172_ALERT_4_G The CIF-Embedded .res File Contains DFIX Records          1 Report
PLAT178_ALERT_4_G The CIF-Embedded .res File Contains SIMU Records          1 Report
PLAT186_ALERT_4_G The CIF-Embedded .res File Contains ISOR Records          2 Report
PLAT188_ALERT_3_G A Non-default SIMU Restraint Value has been used     0.0100 Report
PLAT230_ALERT_2_G Hirshfeld Test Diff for    P015     --C18      .        5.2 s.u.
PLAT232_ALERT_2_G Hirshfeld Test Diff (M-X)  Ag01     --S00X     .        6.3 s.u.
PLAT233_ALERT_4_G Hirshfeld (M-X Solvent)    Sb0E     --F06J     .        9.7 s.u.
PLAT233_ALERT_4_G Hirshfeld (M-X Solvent)    Sb0E     --F06K     .       11.5 s.u.
PLAT233_ALERT_4_G Hirshfeld (M-X Solvent)    Sb0E     --F03W_b   .       11.2 s.u.
PLAT233_ALERT_4_G Hirshfeld (M-X Solvent)    Sb0E     --F06J_b   .       14.8 s.u.
PLAT233_ALERT_4_G Hirshfeld (M-X Solvent)    Sb0E     --F06K_b   .        8.2 s.u.
PLAT299_ALERT_4_G Atom Site Occupancy Constrained at ............         0.5 Check
              F4      F37     C14     C16     C18     C21     C30     C34
              C58     C71     C151    C07X    C07Y    C07R    H14     H16
              H21     H30     H58     H71     H07Y    H151    Sb0E
PLAT301_ALERT_3_G Main Residue  Disorder .............(Resd    1)          3% Note
PLAT302_ALERT_4_G Anion/Solvent/Minor-Residue Disorder (Resd    2)         14% Note
PLAT432_ALERT_2_G Short Inter X...Y Contact  F4       ..C07J     .       2.28 Ang.
                                            1/2+x,1-y,z  =      6_665 Check
PLAT432_ALERT_2_G Short Inter X...Y Contact  F4       ..C157     .       2.79 Ang.
                                            1/2+x,1-y,z  =      6_665 Check
PLAT432_ALERT_2_G Short Inter X...Y Contact  F01H     ..C034     .       2.94 Ang.
                                                  x,y,z  =      1_555 Check
```

```
PLAT432_ALERT_2_G Short Inter X...Y Contact  F055     ..C064      .       2.92 Ang.
                                       1-x,-1/2+y,1/2-z  =      4_545 Check
PLAT432_ALERT_2_G Short Inter X...Y Contact  F066     ..C34       .       2.71 Ang.
                                            1-x,1-y,1-z  =      5_666 Check
PLAT432_ALERT_2_G Short Inter X...Y Contact  F066     ..C157      .       2.81 Ang.
                                            1/2-x,y,1-z  =      2_556 Check
PLAT432_ALERT_2_G Short Inter X...Y Contact  F066     ..C07J      .       2.94 Ang.
                                            1/2-x,y,1-z  =      2_556 Check
PLAT434_ALERT_2_G Short Inter HL..HL Contact F032     ..F05R      .       2.80 Ang.
                                         x,1/2-y,-1/2+z  =      8_554 Check
```

Author Response: F066 is the same atom, and the distance between adjacent molecules formed by symmetrical stacking is very close, so the distance between F066 and F066 is correspondingly small.

```
PLAT434_ALERT_2_G Short Inter HL..HL Contact F066     ..F37       .       2.27 Ang.
                                            1-x,1-y,1-z  =      5_666 Check
```

Author Response: F066 is the same atom, and the distance between adjacent molecules formed by symmetrical stacking is very close, so the distance between F066 and F066 is correspondingly small.

```
PLAT606_ALERT_4_G Solvent Accessible VOID(S) in Structure ........          ! Info
PLAT720_ALERT_4_G Number of Unusual/Non-Standard Labels ..........        401 Note
              Ag01    Sb02    Cu03    Cu04    Cu05    Cu06    Cu07    Cu08
              Cu09    Cu0A    Cu0B    Cu0C    Cu0D    Sb0E    Cu0F    Cu0G
              Cu0H    Cu0I    Cu0J    Cu0K    S0L     S0M     S00N    P00O
              S00P    S00Q    P00R    S00S    S00T    S00U    S00V    S00W
              S00X    S00Y    P00Z    S010    P011    S012    S013    P015
              P016    F017    F018    F019    F01A    F01B    F01C    F01D
              F01E    F01F    F01G    F01H    C01I    H01I    C01J    F01K
              F01L    F01M    F01N    F01O    F01P    C01Q    C01R    C01T
              H01T    C03M    H03M    C041    C023    H023    C02W    H02W
              F01S    C01U    H01U    F01V    F01W    C01Y    C05M    H05M
              C05X    H05X    C05W    C04K    H04K    C02K    H02K    C01Z
              C035    H035    C03Z    H03Z    C05C    C06V    H06V    C06D
              H06D    F020    C022    H022    C025    F026    C027    C01X
              H01X    C024    H024    C03H    C057    H057    C02X    H02X
              C028    H028    C029    H029    C02A    C03F    H03F    C06C
              H06C    C065    C067    H067    C05S    H05S    C02B    C02C
              H02C    C02D    C02E    C05I    H05I    C06Y    H06Y    C05O
              C042    H042    C03S    H03S    C02F    C02G    C02H    H02H
              C02I    C02J    H02J    C02L    H02L    C02M    C06Z    H06Z
              C06H    H06H    C048    C04O    H04O    C05A    H05A    C02N
              H02N    C02O    H02O    C02P    C04H    H04H    C04W    H04W
              C044    C04D    H04D    C03Q    H03Q    C02Q    H02Q    C02R
              C02T    H02T    C02U    F02V    C02Y    H02Y    C02Z    C030
              C031    H031    F032    F033    C034    H034    F036    C038
              H038    F039    C03A    C03C    H03C    C03D    C03E    C021
              H021    C03Y    H03Y    C049    C037    H037    C03X    H03X
              C03G    H03G    C03I    H03I    C03K    H03K    C03L    H03L
              C03O    H03O    C03P    C03T    H03T    F03U    C03V    H03V
              F03W    C040    H040    C046    C04A    H04A    C04C    H04C
```

```
             F04E    C04F    C059    H059    C05H    H05H    C04S    C062
             H062    C05G    H05G    C04G    H04G    C04I    C05J    H05J
             C064    H064    C070    C05F    H05F    C063    H063    C04J
             H04J    C04L    C04M    H04M    C04N    C04P    H04P    C04R
             C04U    H04U    C04V    H04V    C04X    H04X    C04Y    H04Y
             C050    C051    H051    C052    H052    C045    H045    C05B
             C05N    H05N    C04Q    H04Q    C03B    C053    C054    C04Z
             H04Z    C05Y    H05Y    C043    C03J    H03J    C04B    H04B
             F055    C056    H056    C058    H058    C05D    C079    H079
             C07E    H07E    C07U    C07F    H07F    C06T    H06T    C05E
             H05E    C05K    H05K    C05L    C06Q    H06Q    C06A    H06A
             C03N    C069    H069    C047    H047    C05P    C05Q    H05Q
             F05R    C05U    H05U    C05V    H05V    C05Z    H05Z    F060
             F061    F066    C06B    C06E    H06B    H06E    C06F    C071
             H071    C07J    H07J    C07P    H07P    C06I    H06I    C06G
             H06G    C07W    H07W    C06X    C078    H078    C05T    H05T
             C06P    F06J    F06K    C06L    F06M    C06N    H06N    C06O
             H06O    C06U    H06U    C04T    C06W    H06W    C07A    H07A
             C07B    C068    H068    C074    H074    C076    H076    C07C
             C07G    H07G    C07L    C07D    H07D    C07O    H07O    C075
             H075    C07Q    C07T    H07B    H07C    C07X    C07Y    H07Y
             C07R
PLAT779_ALERT_4_G Suspect or Irrelevant (Bond) Angle(s) in CIF ...      27.20 Deg.
             SB0E -F03W -SB0E    1_555   1_555   2_556 ......     #  679 Check
PLAT779_ALERT_4_G Suspect or Irrelevant (Bond) Angle(s) in CIF ...      21.80 Deg.
             SB0E -F06J -SB0E    1_555   1_555   2_556 ......     #  914 Check
PLAT779_ALERT_4_G Suspect or Irrelevant (Bond) Angle(s) in CIF ...      24.30 Deg.
             SB0E -F06K -SB0E    1_555   1_555   2_556 ......     #  915 Check
PLAT794_ALERT_5_G Tentative Bond Valency for Sb02      (V)       .       4.93 Info
PLAT794_ALERT_5_G Tentative Bond Valency for Cu03      (I)       .       1.09 Info
PLAT794_ALERT_5_G Tentative Bond Valency for Cu04      (I)       .       1.03 Info
PLAT794_ALERT_5_G Tentative Bond Valency for Cu06      (I)       .       1.16 Info
PLAT794_ALERT_5_G Tentative Bond Valency for Cu08      (I)       .       1.10 Info
PLAT794_ALERT_5_G Tentative Bond Valency for Cu0B      (I)       .       1.08 Info
PLAT794_ALERT_5_G Tentative Bond Valency for Cu0C      (I)       .       1.07 Info
PLAT794_ALERT_5_G Tentative Bond Valency for Cu0D      (II)      .       1.85 Info
PLAT794_ALERT_5_G Tentative Bond Valency for Cu0J      (I)       .       0.99 Info
PLAT794_ALERT_5_G Tentative Bond Valency for Cu0K      (I)       .       1.01 Info
PLAT860_ALERT_3_G Number of Least-Squares Restraints ............       2803 Note
PLAT868_ALERT_4_G ALERTS Due to the Use of _smtbx_masks Suppressed          ! Info
PLAT883_ALERT_1_G Absent Datum for _atom_sites_solution_primary ..     Please Do !
PLAT933_ALERT_2_G Number of HKL-OMIT Records in Embedded .res File        14 Note
              -1  1  6,   1  1  4,  -1  1  8,  -3  3  6,   2  2 10,  -5  1  2,
              -9  1  2,  -1 11  4,   1  4  5,   0  8  4,   0 13 13,  -2  1  3,
              -4  1  5,  -3 10 17,
PLAT941_ALERT_3_G Average HKL Measurement Multiplicity ..........        2.9 Low
PLAT967_ALERT_5_G Note: Two-Theta Cutoff Value in Embedded .res ..      125.0 Degree
```

---

   1 **ALERT level A** = Most likely a serious problem - resolve or explain
   2 **ALERT level B** = A potentially serious problem, consider carefully
  38 **ALERT level C** = Check. Ensure it is not caused by an omission or oversight
  48 **ALERT level G** = General information/check it is not something unexpected

   2 ALERT type 1 CIF construction/syntax error, inconsistent or missing data

```
43 ALERT type 2 Indicator that the structure model may be wrong or deficient
 7 ALERT type 3 Indicator that the structure quality may be low
26 ALERT type 4 Improvement, methodology, query or suggestion
11 ALERT type 5 Informative message, check
```

It is advisable to attempt to resolve as many as possible of the alerts in all categories. Often the minor alerts point to easily fixed oversights, errors and omissions in your CIF or refinement strategy, so attention to these fine details can be worthwhile. In order to resolve some of the more serious problems it may be necessary to carry out additional measurements or structure refinements. However, the purpose of your study may justify the reported deviations and the more serious of these should normally be commented upon in the discussion or experimental section of a paper or in the "special_details" fields of the CIF. checkCIF was carefully designed to identify outliers and unusual parameters, but every test has its limitations and alerts that are not important in a particular case may appear. Conversely, the absence of alerts does not guarantee there are no aspects of the results needing attention. It is up to the individual to critically assess their own results and, if necessary, seek expert advice.

Publication of your CIF in IUCr journals

A basic structural check has been run on your CIF. These basic checks will be run on all CIFs submitted for publication in IUCr journals (*Acta Crystallographica*, *Journal of Applied Crystallography*, *Journal of Synchrotron Radiation*); however, if you intend to submit to *Acta Crystallographica Section C* or *E* or *IUCrData*, you should make sure that full publication checks are run on the final version of your CIF prior to submission.

Publication of your CIF in other journals

Please refer to the *Notes for Authors* of the relevant journal for any special instructions relating to CIF submission.

PLATON version of 02/02/2025; check.def file version of 02/02/2025

Datablock 1 - ellipsoid plot

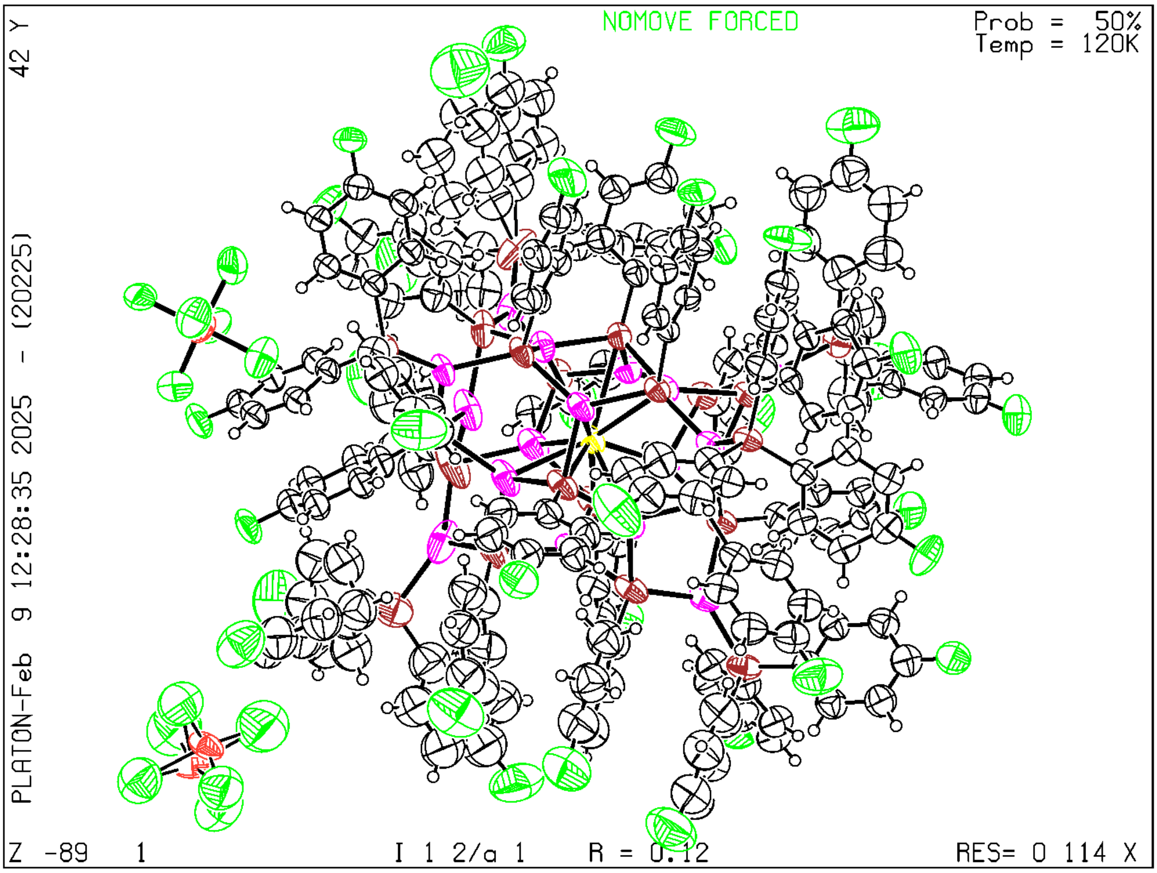

Supplement: nwag053_Supplemental_Files [file nwag053_supplemental_files.zip › Checkcif of Ag1Cu17.pdf]
