# Supplementary material for: Spatial and temporal control over photoresponsive nanoclusters
Source: Natl Sci Rev. 2026 Jan 28;13(5):nwag053. doi: 10.1093/nsr/nwag053 (PMC12912717; doi:10.1093/nsr/nwag053)

checkCIF/PLATON report

You have not supplied any structure factors. As a result the full set of tests cannot be run.

THIS REPORT IS FOR GUIDANCE ONLY. IF USED AS PART OF A REVIEW PROCEDURE FOR PUBLICATION, IT SHOULD NOT REPLACE THE EXPERTISE OF AN EXPERIENCED CRYSTALLOGRAPHIC REFEREE.

No syntax errors found. CIF dictionary Interpreting this report

Datablock: 1

```
Bond precision:   C-C = 0.0179 A          Wavelength=1.54186

Cell:              a=36.8203(4)     b=32.2641(4)     c=37.2014(5)
                   alpha=90         beta=90          gamma=90
Temperature:       120 K
```

	Calculated	Reported
Volume	44194.3(9)	44194.3(9)
Space group	P b c a	P b c a
Hall group	-P 2ac 2ab	-P 2ac 2ab
Moiety formula	C198 H132 Cu18 F33 P6 S15, F6 Sb [+ solvent]	F6 Sb, C198 H132 Cu18 F33 P6 S15
Sum formula	C198 H132 Cu18 F39 P6 S15 Sb [+ solvent]	C198 H132 Cu18 F39 P6 S15 Sb
Mr	5184.38	5184.22
Dx,g cm-3	1.558	1.558
Z	8	8
Mu (mm-1)	5.179	5.179
F000	20592.0	20592.0
F000’	20412.06	
h,k,lmax	43,37,43	43,37,43
Nref	37601	36510
Tmin,Tmax	0.453,0.460	0.356,0.461
Tmin’	0.309	

```
Correction method= # Reported T Limits: Tmin=0.356 Tmax=0.461
AbsCorr = MULTI-SCAN

Data completeness= 0.971          Theta(max)= 64.997
```

R(reflections)= 0.0823(18086)

wR2(reflections)= 0.2357(36510)

S = 0.888 Npar= 2497

The following ALERTS were generated. Each ALERT has the format
test-name_ALERT_alert-type_alert-level.
Click on the hyperlinks for more details of the test.

Alert level C

```
ABSTY02_ALERT_1_C  An _exptl_absorpt_correction_type has been given without
            a literature citation. This should be contained in the
            _exptl_absorpt_process_details field.
            Absorption correction given as multi-scan
RINTA01_ALERT_3_C  The value of Rint is greater than 0.12
            Rint given   0.134
THETM01_ALERT_3_C  The value of sine(theta_max)/wavelength is less than 0.590
            Calculated sin(theta_max)/wavelength =    0.5878
PLAT020_ALERT_3_C The Value of Rint is Greater Than 0.12 .........      0.134 Report
PLAT029_ALERT_3_C _diffrn_measured_fraction_theta_full value Low .      0.971 Why?
PLAT213_ALERT_2_C Atom F1              has ADP max/min Ratio .....        3.2 prolat
PLAT213_ALERT_2_C Atom F04L            has ADP max/min Ratio .....        3.4 prolat
PLAT220_ALERT_2_C NonSolvent   Resd 1  C   Ueq(max)/Ueq(min) Range        3.7 Ratio
PLAT234_ALERT_4_C Large Hirshfeld Difference F03M     --C06R     .       0.18 Ang.
PLAT234_ALERT_4_C Large Hirshfeld Difference Sb01     --F03W     .       0.16 Ang.
PLAT242_ALERT_2_C Low    'MainMol' Ueq as Compared to Neighbors of       C05K Check
PLAT242_ALERT_2_C Low    'MainMol' Ueq as Compared to Neighbors of       C07O Check
PLAT242_ALERT_2_C Low    'MainMol' Ueq as Compared to Neighbors of       C07R Check
PLAT242_ALERT_2_C Low    'MainMol' Ueq as Compared to Neighbors of       C06E Check
PLAT242_ALERT_2_C Low    'MainMol' Ueq as Compared to Neighbors of       C06R Check
PLAT242_ALERT_2_C Low    'MainMol' Ueq as Compared to Neighbors of       C07X Check
PLAT242_ALERT_2_C Low    'MainMol' Ueq as Compared to Neighbors of       C07E Check
PLAT242_ALERT_2_C Low    'MainMol' Ueq as Compared to Neighbors of       C07M Check
PLAT242_ALERT_2_C Low    'MainMol' Ueq as Compared to Neighbors of       C08C Check
PLAT244_ALERT_4_C Low    'Solvent' Ueq as Compared to Neighbors of       Sb01 Check
PLAT260_ALERT_2_C Large Average Ueq of Residue Including      Sb01      0.115 Check
PLAT334_ALERT_2_C Small <C-C> Benzene Dist.   C01X     -C047     .       1.37 Ang.
PLAT342_ALERT_3_C Low Bond Precision on  C-C Bonds ...............    0.01788 Ang.
```

Alert level G

```
PLAT003_ALERT_2_G Number of Uiso or Uij Restrained non-H Atoms ...        244 Report
PLAT042_ALERT_1_G Calc. and Reported MoietyFormula Strings  Differ     Please Check
              Calc: C198 H132 Cu18 F33 P6 S15, F6 Sb
              Rep.: F6 Sb, C198 H132 Cu18 F33 P6 S15
PLAT072_ALERT_2_G SHELXL First  Parameter in WGHT  Unusually Large       0.13 Report
PLAT178_ALERT_4_G The CIF-Embedded .res File Contains SIMU Records          1 Report
PLAT186_ALERT_4_G The CIF-Embedded .res File Contains ISOR Records          2 Report
PLAT188_ALERT_3_G A Non-default SIMU Restraint Value has been used     0.0100 Report
PLAT230_ALERT_2_G Hirshfeld Test Diff for    S00U     --C10      .        5.4 s.u.
PLAT232_ALERT_2_G Hirshfeld Test Diff (M-X)  Cu0H     --S00U     .        5.3 s.u.
PLAT300_ALERT_4_G Atom Site Occupancy of F6         Constrained at        0.5 Check
PLAT300_ALERT_4_G Atom Site Occupancy of F077       Constrained at        0.5 Check
PLAT300_ALERT_4_G Atom Site Occupancy of C8         Constrained at        0.5 Check
PLAT300_ALERT_4_G Atom Site Occupancy of C9         Constrained at        0.5 Check
```

```
PLAT300_ALERT_4_G Atom Site Occupancy of C10        Constrained at        0.5 Check
PLAT300_ALERT_4_G Atom Site Occupancy of C15        Constrained at        0.5 Check
PLAT300_ALERT_4_G Atom Site Occupancy of C19        Constrained at        0.5 Check
PLAT300_ALERT_4_G Atom Site Occupancy of C31        Constrained at        0.5 Check
PLAT300_ALERT_4_G Atom Site Occupancy of C082       Constrained at        0.5 Check
PLAT300_ALERT_4_G Atom Site Occupancy of C07I       Constrained at        0.5 Check
PLAT300_ALERT_4_G Atom Site Occupancy of C07L       Constrained at        0.5 Check
PLAT300_ALERT_4_G Atom Site Occupancy of C07W       Constrained at        0.5 Check
PLAT300_ALERT_4_G Atom Site Occupancy of C087       Constrained at        0.5 Check
PLAT300_ALERT_4_G Atom Site Occupancy of C04X       Constrained at        0.5 Check
PLAT300_ALERT_4_G Atom Site Occupancy of H8         Constrained at        0.5 Check
PLAT300_ALERT_4_G Atom Site Occupancy of H9         Constrained at        0.5 Check
PLAT300_ALERT_4_G Atom Site Occupancy of H15        Constrained at        0.5 Check
PLAT300_ALERT_4_G Atom Site Occupancy of H31        Constrained at        0.5 Check
PLAT300_ALERT_4_G Atom Site Occupancy of H082       Constrained at        0.5 Check
PLAT300_ALERT_4_G Atom Site Occupancy of H07I       Constrained at        0.5 Check
PLAT300_ALERT_4_G Atom Site Occupancy of H07W       Constrained at        0.5 Check
PLAT300_ALERT_4_G Atom Site Occupancy of H087       Constrained at        0.5 Check
PLAT301_ALERT_3_G Main Residue  Disorder .............(Resd    1)          3% Note
PLAT410_ALERT_2_G Short Intra H...H Contact  H8       ..H061     .        1.98 Ang.
                                                      x,y,z  =      1_555 Check
PLAT410_ALERT_2_G Short Intra H...H Contact  H08E     ..H07W     .        2.05 Ang.
                                                      x,y,z  =      1_555 Check
PLAT410_ALERT_2_G Short Intra H...H Contact  H08E     ..H087     .        1.93 Ang.
                                                      x,y,z  =      1_555 Check
PLAT432_ALERT_2_G Short Inter X...Y Contact  F6       ..C05L     .        2.85 Ang.
                                              1/2+x,y,3/2-z  =      6_657 Check
PLAT432_ALERT_2_G Short Inter X...Y Contact  F04D     ..C06X     .        2.91 Ang.
                                                      x,y,z  =      1_555 Check
PLAT432_ALERT_2_G Short Inter X...Y Contact  F04P     ..C03H     .        2.93 Ang.
                                             -1/2+x,y,3/2-z  =      6_557 Check
PLAT434_ALERT_2_G Short Inter HL..HL Contact F01M     ..F01M     .        2.72 Ang.
                                                1-x,1-y,1-z  =      5_666 Check
PLAT434_ALERT_2_G Short Inter HL..HL Contact F01O     ..F04L     .        2.80 Ang.
                                              1/2-x,1/2+y,z  =      8_665 Check
PLAT434_ALERT_2_G Short Inter HL..HL Contact F01S     ..F077     .        2.82 Ang.
                                             -1/2+x,y,3/2-z  =      6_557 Check
PLAT434_ALERT_2_G Short Inter HL..HL Contact F02T     ..F02Y     .        2.81 Ang.
                                             -1/2+x,y,3/2-z  =      6_557 Check
PLAT606_ALERT_4_G Solvent Accessible VOID(S) in Structure ........           ! Info
PLAT720_ALERT_4_G Number of Unusual/Non-Standard Labels ..........         407 Note
              Sb01    Cu02    Cu03    Cu04    Cu05    Cu06    Cu07    Cu08
              Cu09    Cu0A    Cu0B    Cu0C    Cu0D    Cu0E    Cu0F    Cu0G
              Cu0H    Cu0I    Cu0J    S00K    S00L    S00N    S00O    S00P
              S00Q    S00R    S00S    P00T    S00U    S00V    S00W    S00X
              S00Y    S00Z    S010    P011    P012    P013    P014    P016
              F01A    F01B    F01C    F01D    F01E    F01F    F01G    F01I
              F01J    F01L    F01M    F01N    F01O    F01P    C01Q    F01R
              F01S    F01U    F01V    F01W    C01X    C01Z    F020    C021
              F022    C023    H023    C024    H024    C025    H025    C026
              F027    C028    C029    C02A    H02A    C02B    H02B    C02C
              C02D    H02D    C02E    H02E    C02F    C02G    H02G    C02H
              C02I    H02I    C02J    C02L    C02M    C02N    C02O    H02O
              C02P    H02P    C02Q    C02R    C02S    H02S    F02T    C02U
              H02U    C02V    H02V    C02W    C02X    H02X    F02Y    C02Z
              H02Z    C030    H030    C031    C032    H032    C033    C034
              C035    C036    C037    H037    C038    C039    H039    C03A
```

```
               H03A    C03B    H03B    C03C    H03C    C03D    C03E    H03E
               C03F    H03F    C03G    H03G    C03H    C03J    H03J    C03K
               H03K    C03L    H03L    F03M    C03N    H03N    C03O    H03O
               C03P    C03Q    H03Q    C03R    H03R    C03S    C03T    H03T
               C03U    H03U    C03V    H03V    F03W    C03X    H03X    C03Y
               H03Y    C03Z    H03Z    C040    H040    C041    H041    C042
               C043    C044    C045    C046    C047    H047    C048    H048
               C049    H049    C04A    H04A    C04B    C04C    H04C    F04D
               C04E    H04E    C04F    H04F    C04G    C04H    H04H    C04I
               C04J    H04J    F04K    F04L    C04M    C04N    H04N    C04O
               H04O    F04P    F04Q    C04R    H04R    C04S    H04S    C04T
               H04T    C04U    H04U    C04V    C04W    C04Y    H04Y    C050
               C052    C053    C054    F055    C056    H056    C057    H057
               C058    H058    C059    H059    C05A    H05A    C05B    H05B
               C05C    C05D    H05D    C05F    H05F    C05G    H05G    C05H
               H05H    C05J    C05K    C05L    C05M    H05M    C05N    H05N
               C05O    H05O    C05P    H05P    C05R    H05R    C05S    H05S
               C05U    H05U    F05V    F05W    C05X    H05X    C05Y    C05Z
               H05Z    C060    H060    C061    H061    C062    H062    C063
               H063    C064    C05T    H05T    C08E    H08E    C07O    C06M
               H06M    C06S    H06S    C065    H065    C066    H066    C067
               H067    C068    H068    C069    H069    C06A    C06V    H06V
               C07Q    H07Q    C07R    C07Y    H07Y    C07P    H07P    C06B
               H06B    C06C    H06C    C06D    H06D    C06E    C06F    H06F
               C06G    H06G    C06H    C06I    H06I    C06J    H06J    C06K
               H06K    C06L    H06L    C06N    H06N    F06O    C06P    H06P
               C06Q    H06Q    C06R    C06T    H06T    C06U    H06U    C076
               C07N    H07N    C086    H086    C07X    C081    H081    C06W
               H06W    C06X    H06X    C06Y    H06Y    C070    H070    C071
               H071    C072    H072    C073    C074    H074    C075    F077
               C078    H078    C079    H079    C07B    F07C    C07D    H07D
               C07E    C07F    C07G    H07G    C07H    C07J    H07J    C07M
               C07S    H07S    C07T    H07T    C07U    H07U    C07V    H07V
               C082    H082    C07I    H07I    C07L    C07W    H07W    C087
               H087    C04X    C08A    H08A    C08B    H08B    C08C
PLAT794_ALERT_5_G Tentative Bond Valency for Sb01      (V)       .       5.96 Info
PLAT794_ALERT_5_G Tentative Bond Valency for Cu02      (I)       .       0.25 Info
PLAT794_ALERT_5_G Tentative Bond Valency for Cu04      (I)       .       0.92 Info
PLAT794_ALERT_5_G Tentative Bond Valency for Cu05      (I)       .       1.16 Info
PLAT794_ALERT_5_G Tentative Bond Valency for Cu06      (I)       .       0.91 Info
PLAT794_ALERT_5_G Tentative Bond Valency for Cu07      (I)       .       1.03 Info
PLAT794_ALERT_5_G Tentative Bond Valency for Cu08      (I)       .       1.06 Info
PLAT794_ALERT_5_G Tentative Bond Valency for Cu09      (I)       .       0.94 Info
PLAT794_ALERT_5_G Tentative Bond Valency for Cu0A      (I)       .       1.08 Info
PLAT794_ALERT_5_G Tentative Bond Valency for Cu0B      (I)       .       1.08 Info
PLAT794_ALERT_5_G Tentative Bond Valency for Cu0D      (I)       .       0.96 Info
PLAT794_ALERT_5_G Tentative Bond Valency for Cu0E      (I)       .       1.06 Info
PLAT794_ALERT_5_G Tentative Bond Valency for Cu0F      (II)      .       1.84 Info
PLAT794_ALERT_5_G Tentative Bond Valency for Cu0G      (I)       .       0.94 Info
PLAT794_ALERT_5_G Tentative Bond Valency for Cu0I      (I)       .       1.11 Info
PLAT794_ALERT_5_G Tentative Bond Valency for Cu0J      (I)       .       1.09 Info
PLAT860_ALERT_3_G Number of Least-Squares Restraints ............       2814 Note
PLAT868_ALERT_4_G ALERTS Due to the Use of _smtbx_masks Suppressed          ! Info
PLAT933_ALERT_2_G Number of HKL-OMIT Records in Embedded .res File          7 Note
                7  2  1,   4  2  3,   1  4  7,   4  2  5,   2  3 11,   8  0  0,
                1  1  6,
```

```
 0 ALERT level A = Most likely a serious problem - resolve or explain
 0 ALERT level B = A potentially serious problem, consider carefully
23 ALERT level C = Check. Ensure it is not caused by an omission or oversight
62 ALERT level G = General information/check it is not something unexpected

 2 ALERT type 1 CIF construction/syntax error, inconsistent or missing data
29 ALERT type 2 Indicator that the structure model may be wrong or deficient
 8 ALERT type 3 Indicator that the structure quality may be low
30 ALERT type 4 Improvement, methodology, query or suggestion
16 ALERT type 5 Informative message, check
```

It is advisable to attempt to resolve as many as possible of the alerts in all categories. Often the minor alerts point to easily fixed oversights, errors and omissions in your CIF or refinement strategy, so attention to these fine details can be worthwhile. In order to resolve some of the more serious problems it may be necessary to carry out additional measurements or structure refinements. However, the purpose of your study may justify the reported deviations and the more serious of these should normally be commented upon in the discussion or experimental section of a paper or in the "special_details" fields of the CIF. checkCIF was carefully designed to identify outliers and unusual parameters, but every test has its limitations and alerts that are not important in a particular case may appear. Conversely, the absence of alerts does not guarantee there are no aspects of the results needing attention. It is up to the individual to critically assess their own results and, if necessary, seek expert advice.

Publication of your CIF in IUCr journals

A basic structural check has been run on your CIF. These basic checks will be run on all CIFs submitted for publication in IUCr journals (*Acta Crystallographica*, *Journal of Applied Crystallography*, *Journal of Synchrotron Radiation*); however, if you intend to submit to *Acta Crystallographica Section C* or *E* or *IUCrData*, you should make sure that full publication checks are run on the final version of your CIF prior to submission.

Publication of your CIF in other journals

Please refer to the *Notes for Authors* of the relevant journal for any special instructions relating to CIF submission.

PLATON version of 13/12/2023; check.def file version of 13/12/2023

Datablock 1 - ellipsoid plot

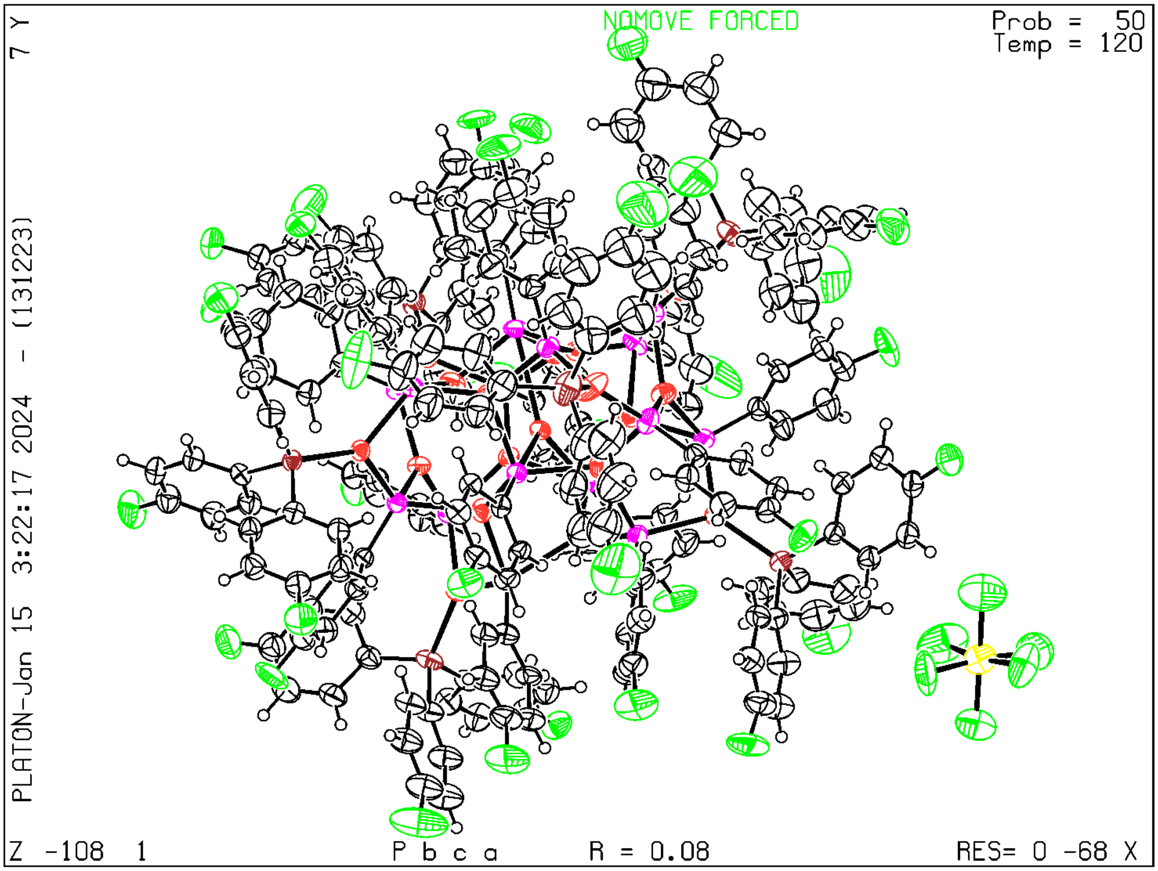

Supplement: nwag053_Supplemental_Files [file nwag053_supplemental_files.zip › Checkcif of Cu18.pdf]
