# Supplementary material for: Spatial and temporal control over photoresponsive nanoclusters
Source: Natl Sci Rev. 2026 Jan 28;13(5):nwag053. doi: 10.1093/nsr/nwag053 (PMC12912717; doi:10.1093/nsr/nwag053)
Supplement: nwag053_Supplemental_Files [file nwag053_supplemental_files.zip › Supplementary data.pdf]

# Spatial and Temporal Control over Photoresponsive Nanoclusters

Ying Xu[1,†], Mengfan Chang[1,†], Hao Li[3], Ning Zhang[4], Siqi Li[4], Pu Wang[2,*], Yong Pei[2,*], Xi Kang[1,*] and Manzhou Zhu[1,*]

[1]Department of Chemistry and Centre for Atomic Engineering of Advanced Materials, Key Laboratory of Structure and Functional Regulation of Hybrid Materials of Ministry of Education, Anhui Province Key Laboratory of Chemistry for Inorganic/Organic Hybrid Functionalized Materials, Anhui University, Hefei, 230601, China.
[2]Department of Chemistry, Key Laboratory of Environmentally Friendly Chemistry and Applications of Ministry of Education, Xiangtan University, Xiangtan, 411105, P. R. China.
[3]School of Materials and Chemical Engineering, Anhui Jianzhu University, Hefei, 230601, China.
[4]State Key Laboratory of Opto-Electronic Information Acquisition and Protection Technology, Anhui University, Hefei, 230601, China.

E-mail: zmz@ahu.edu.cn (M.Z.); kangxi_chem@ahu.edu.cn (X.K.); ypei2@xtu.edu.cn (Y.P.); 90wangpu@xtu.edu.cn (P.W.)

[†]These authors contributed equally to this work.

*This Supporting Information file includes:*

1. Experimental Methods
2. Computational Details
3. Scheme S1 and Figures S1-S31
4. Crystal Data and Structure Refinement

## 1. Experimental Methods

### Chemical Materials

All the following reagents and solvents were commercially available and all of them were used without further purification, including silver nitrate ($AgNO_3$, 99%), cuprous chloride (CuCl, 99%), tris(4-fluorophenyl)phosphine ($P(Ph^pF)_3$, 98%), 4-fluorothiophenol ($HSPh^pF$, 97%), sodium hexafluoroantimonate ($NaSbF_6$, 98%), sodium borohydride ($NaBH_4$, 98%), sodium borodeuteride ($NaBD_4$), dichloromethane ($CH_2Cl_2$, HPLC grade), methanol (MeOH, HPLC grade), *n*-hexane (HPLC grade), trichloromethane ($CH_3Cl$, HPLC grade), and acetonitrile ($CH_3CN$ HPLC grade).

### Preparation of the precursor Ag-$P(Ph^pF)_3$ complexes

8.5 mg of $AgNO_3$ was dissolved in 8.5 mL of $CH_3OH$, and 20 mg of $P(Ph^pF)_3$ (dissolved in 1.5 mL of $CH_2Cl_2$) was added. The colorless and transparent Ag-$P(Ph^pF)_3$ complex solution was obtained.

### Preparation of the $Cu_{18}H_2(SPh^pF)_{15}[P(Ph^pF)_3]_6(SbF_6)_1$

150 mg of CuCl was dissolved in a mixed solvent of 5 mL of $CH_3CN$ and 45 mL of $CH_2Cl_2$ under ice-bath conditions. Then, 120 μL of $HSPh^pF$ was added, resulting in a colorless and transparent solution. After 1 hour, 360 mg of $P(Ph^pF)_3$ was added. After 40 minutes, a freshly prepared aqueous solution of $NaBH_4$ (180 mg/5 mL) was added to the solution, and the solution color changed to brown. The reaction was proceed for another 8 hours. The obtained solution was centrifuged, and the supernatant was collected. Then, 10 mg of $NaSbF_6$ (dissolved in MeOH) was added to the supernatant. The crude product was purified by rotary evaporation and was further washed with *n*-hexane 3 to 4 times. The yellow crystals were obtained with a $CH_2Cl_2$/*n*-hexane liquid/liquid crystallization pattern at 4 °C. After a week, yellow crystals were collected, and the crystal structure of **$Cu_{18}$** was determined. The absolute yield was 18.0 mg, and the percentage yield was calculated to be 4.63% based on the S element for the **$Cu_{18}$** nanocluster.

### Preparation of the $Cu_{18}D_2(SPh^pF)_{15}[P(Ph^pF)_3]_6(SbF_6)_1$

The preparation of **$Cu_{18}$-D** was similar to that of the **$Cu_{18}$** nanocluster, except that the $NaBH_4$ was replaced by $NaBD_4$ with the same molar quantity. The absolute yield was 17.1 mg, and the percentage yield was calculated to be 4.39% based on the S element for the **$Cu_{18}$-D** nanocluster.

### Preparation of the $Ag_1Cu_{17}H_2(SPh^pF)_{15}[P(Ph^pF)_3]_6(SbF_6)_1$

**Method 1 (in-situ synthetic approach):** the preparation of **$Ag_1Cu_{17}$** was similar to that of the **$Cu_{18}$** nanocluster, except that 30 mg of $AgNO_3$ (dissolved in 5 mL of methanol) was added before the addition of $P(Ph^pF)_3$. The absolute yield was 70.2 mg, and the percentage yield was calculated to be 17.88% based on the S element for the **$Ag_1Cu_{17}$** nanocluster.

**Method 2 (doping approach):** 2 mg of **$Cu_{18}$** crystals were dissolved in 2 mL of $CH_2Cl_2$, and 300 μL of Ag-$P(Ph^pF)_3$ complex solution was added. After 5 minutes, the solution had evaporated, and the product was obtained. Yellow block crystals of **$Ag_1Cu_{17}$** were obtained with a $CH_2Cl_2$/*n*-hexane liquid/liquid crystallization pattern at 4 °C for a week. The absolute yield was 1.8 mg, and the percentage yield was calculated to be 89.11% based on **$Cu_{18}$** for the **$Cu_{18}$-D** nanocluster.

### Preparation of the $Ag_1Cu_{17}D_2(SPh^pF)_{15}[P(Ph^pF)_3]_6(SbF_6)_1$

**Method 1 (in-situ synthetic approach):** the preparation of **$Ag_1Cu_{17}$-D** was similar to that of the **$Ag_1Cu_{17}$**

nanocluster, except that the $NaBH_4$ was replaced by $NaBD_4$ with the same molar quantity. The absolute yield was 68.7 mg, and the percentage yield was calculated to be 17.49% based on the S element for the **$Ag_1Cu_{17}$-D** nanocluster.

**Method 2 (doping approach):** the preparation of **$Ag_1Cu_{17}$-D** was similar to that of the **$Ag_1Cu_{17}$** nanocluster with the doping approach, except that **$Cu_{18}$** crystals were replaced by **$Cu_{18}$-D**. The absolute yield was 1.8 mg, and the percentage yield was calculated to be 89.11% based on the S element for the **$Cu_{18}$-D** nanocluster.

**Transformation from $Cu_{18}H_2(SPh^pF)_{15}[P(Ph^pF)_3]_6(SbF_6)_1$ to $\{Cl_1Cu_{14}(SPh^pF)_{12}[P(Ph^pF)_3]_6\}^+$**

The as-obtained **$Cu_{18}$** crystals (2 mg) were dissolved in 3 mL of $CH_2Cl_2$, and the solution was exposed to ultraviolet light (365 nm, 30 W). After 240 seconds, the color of the solution changed from yellow to orange, demonstrating the generation of **$Cu_{14}$**. The final supernatant was centrifuged and washed several times with *n*-hexane.

**Transformation from $Ag_1Cu_{17}H_2(SPh^pF)_{15}[P(Ph^pF)_3]_6(SbF_6)_1$ to $\{Cl_1Cu_{14}(SPh^pF)_{12}[P(Ph^pF)_3]_6\}^+$**

The as-obtained **$Ag_1Cu_{17}$** crystals (2 mg) were dissolved in 3 mL of $CH_2Cl_2$, and the solution was exposed to ultraviolet light (365 nm, 30 W). After 150 seconds, the color of the solution changed from yellow to red, and the **$Cu_{14}$** nanocluster was obtained. The final supernatant was centrifuged and washed several times with *n*-hexane.

**Parameters of the light source**

The photoirradiation conversion experiments were conducted using a 365 nm ultraviolet lamp with a power of 30 W. The light was directed onto the sample at a distance of 3 cm, covering an illumination area of 3.80 $cm^2$, which corresponds to a light intensity of 7.89 $W/cm^2$.

**Single-crystal X-ray diffraction**

The data collection for single-crystal X-ray diffraction (SC-XRD) of all nanocluster crystal samples was carried out on Stoe Stadivari diffractometer under nitrogen flow, using graphite-monochromatized Cu Kα radiation (λ = 1.54186 Å). The structure was solved by direct methods and refined with full-matrix least squares on $F^2$ using the SHELXTL software package. All non-hydrogen atoms were refined anisotropically, and all the hydrogen atoms were set in geometrically calculated positions and refined isotropically using a riding model. All crystal structures were treated with PLATON SQUEEZE. The diffuse electron densities from these residual solvent molecules were removed. All C atoms were refined anisotropically with SIMU 0.01 0.02 2 $C and ISOR 0.01 0.02 $C. All F atoms were refined with ISOR 0.01 0.02 $F. The benzene rings were treated with AFIX 66. Part of P ligands of $NaSbF_6$ were refined as disordered.

The CCDC numbers of **$Cu_{18}$** and **$Ag_1Cu_{17}$** are 2422710 and 2422709, respectively. These data can be obtained free of charge from The Cambridge Crystallographic Data Centre via www.ccdc.cam.ac.uk/data_request/cif.

**Characterizations**

The optical absorption (UV-vis) spectra were performed on a Lambda 465 UV-VIS spectrophotometer. And the quartz cuvette used for the test was 1.25 cm × 1.25 cm × 6 cm in size.

The electrospray ionization mass (ESI-MS) measurements were performed on Waters XEVO G2-XS QT

of the mass spectrometer. The sample was directly infused into the chamber at 40 μl/min. For preparing the ESI samples, the crystals were dissolved in $CH_2Cl_2$ and diluted (v/v=1:1) by $CH_3OH$.

The X-ray photoelectron spectroscopy (XPS) measurements were performed on an ESCALAB 250Xi XPS spectrometer.

The Nuclear Magnetic Resonance (NMR) measurements were carried out on JEOL JNM ECZ600R at 300K.

The micro-nano processing system employs femtosecond laser (MONACO 517) in combination with the Roban fs laser micro-nano processing system and the displacement platform (Newport, XPS-D type). The laser system operates at a wavelength of 517 nm with a repetition rate of 125 kHz. The nominal optical output power is 4 mW, and a 99% beam attenuation is applied during processing, resulting in a laser energy density of 40.8 mJ/cm$^2$.

## 2. Computational Methods

In the determine the locations of two hydrides in $Cu_{18}H_2(SPh^pF)_{15}[P(Ph^pF)_3]_6$ cluster, the $DMol^3$ 8.0 software [1-2] was employed. DFT calculations were performed to optimize the different configurations of $Cu_{18}H_2(SPh^pF)_{15}[P(Ph^pF)_3]_6$ and $Ag_1Cu_{17}H_2(SPh^pF)_{15}[P(Ph^pF)_3]_6$ clusters. The Perdew-Burke-Ernzerhof (PBE) exchange-correlation functional [3], DFT-based relativistic semi-core pseudopotential (DSPP) [4], double-numerical plus d-polarization function (DND) basis sets, and the Tkatchenko-Scheffler (TS) dispersion correction were adopted [5]. The convergence criteria of the geometric optimization was set to $1.0\times10^{-5}$ Hartree for the energy change, $2.0\times10^{-3}$ Hartree/Å for the gradient, and $5.0\times10^{-3}$ Å for the displacement. The diagrams of frontier molecular orbitals of **$Cu_{18}$** and **$Ag_1Cu_{17}$** clusters were also performed using the $DMol^3$ programs.

In the analysis of the interatomic bond lengths of **$Cu_{18}$** and **$Ag_1Cu_{17}$** clusters, the Amsterdam Density Functional (ADF 2016) [6] package was used. The DFT and time-dependent density functional theory (TDDFT) calculations were performed to optimize the ground-state and excited-state geometry structures of **$Cu_{18}$** and **$Ag_1Cu_{17}$** clusters. The BP86 [7-8] exchange correlation functional and an all-electron STO-type basis set of double-ζ (DZ) were used. Scalar relativistic effects were included by utilizing the zeroth-order regular approximation (ZORA) [9]. The energy and gradient convergence criteria were tightened to $1 \times 10^{-4}$ and $1 \times10^{-3}$ to obtain well converged geometries.

**References**


1. Delley B. An all-electron numerical method for solving the local density functional for polyatomic molecules. *J Chem Phys* 1990; **92**: 508-517.

2. Delley B. From molecules to solids with the $DMol^3$ approach. *J Chem Phys* 2000; **113**: 7756-7764.

3. Perdew JP, Burke K, Ernzerhof M. Generalized Gradient Approximation Made Simple. *Phys Rev Lett* 1996; **77**: 3865.

4. Delley B. Ground-State Enthalpies: Evaluation of Electronic Structure Approaches with Emphasis on the Density Functional Method. *J Phys Chem A* 2006; **110**: 13632-13639.

5. Tkatchenko A, Scheffler M. Accurate Molecular Van Der Waals Interactions from Ground-State Electron Density and Free-Atom Reference Data. *Phys Rev Lett* 2009; **102**: 073005.

6. Velde Gte, Bickelhaupt FM, Baerends EJ et al. Chemistry with ADF. *J Comput Chem* 2001; **22**: 931-967.

7. Becke AD. Density-functional exchange-energy approximation with correct asymptotic behavior. *Phys Rev A* 1988; **38**: 3098-3100.

8. Perdew JP. Density-functional approximation for the correlation energy of the inhomogeneous electron gas. *Phys Rev B* 1986; **33**: 8822-8824.

9. Lenthe Ev, Baerends EJ, Snijders JG. Relativistic regular two-component Hamiltonians. *J Chem Phys* 1993; **99**: 4597-4610.

## 3. Scheme S1 and Figures S1-S31

**Scheme S1.** (A) Preparation method for the **$Cu_{18}$** nanocluster. (B) Preparation method for the **$Cu_{18}$-D** nanocluster. (C) Preparation method for the **$Ag_1Cu_{17}$** nanocluster via the alloying approach. (D) Preparation method for the **$Ag_1Cu_{17}$** nanocluster via the one-pot synthetic approach.

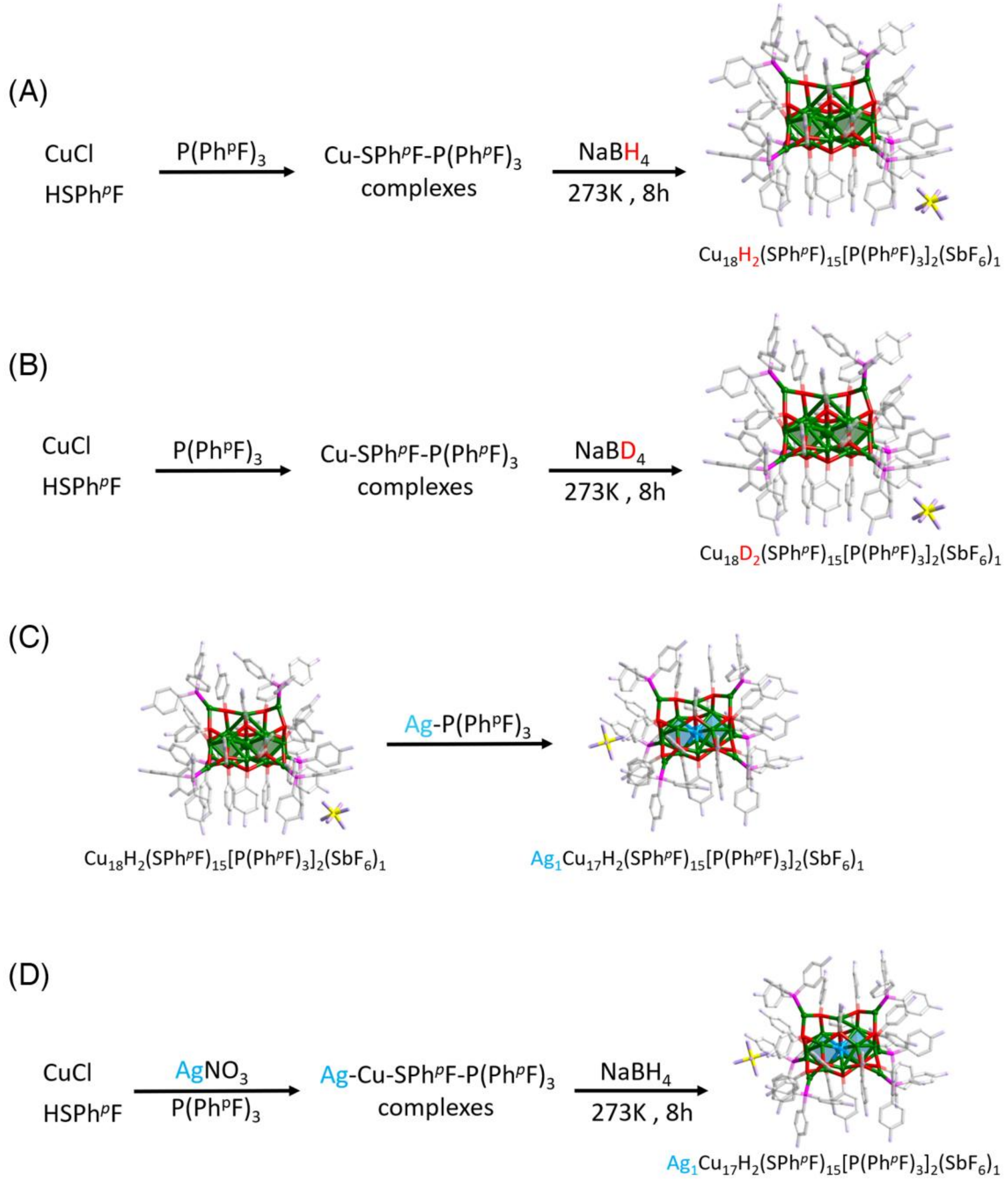

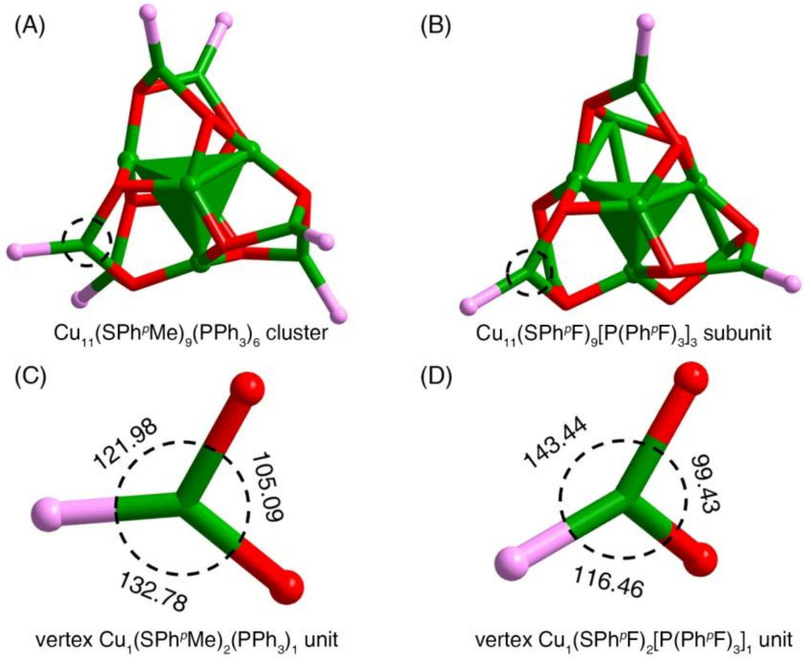


**Figure S1.** Structural comparison between (A) the overall structure of the $Cu_{11}(SPh^{p}Me)_9(PPh_3)_6$ nanocluster and (B) the $Cu_{11}(SPh^{p}F)_9[P(Ph^{p}F)_3]_3$ subunit in **$Cu_{18}$**. (C) The vertex $Cu_1(SPh^{p}Me)_2(PPh_3)_1$ unit of the $Cu_{11}(SPh^{p}Me)_9(PPh_3)_6$ nanocluster. (D) The vertex $Cu_1(SPh^{p}F)_2[P(Ph^{p}F)_3]_1$ of the **$Cu_{18}$** nanocluster. Color labels: green, Cu; magenta, P; red, S. For clarity, all C, F and H atoms are omitted.

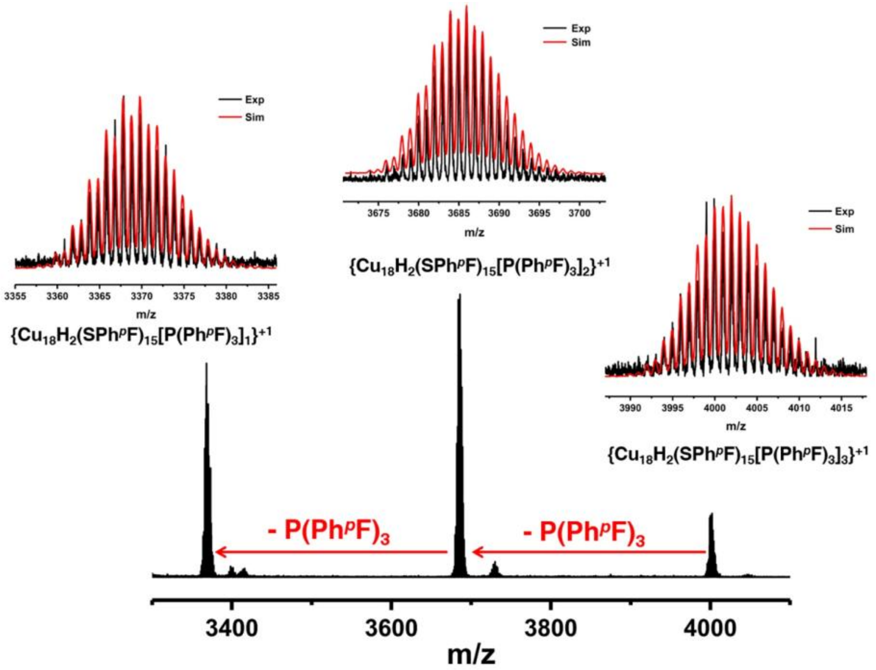


**Figure S2.** ESI-MS result of the **$Cu_{18}$** nanocluster. Three mass signals were detected, including $\{Cu_{18}H_2(SPh^{p}F)_{15}[P(Ph^{p}F)_3]_1\}^{1+}$, $\{Cu_{18}H_2(SPh^{p}F)_{15}[P(Ph^{p}F)_3]_2\}^{1+}$, and $\{Cu_{18}H_2(SPh^{p}F)_{15}[P(Ph^{p}F)_3]_3\}^{1+}$. Insets: experimental (black lines) and calculated (red lines) isotope patterns.

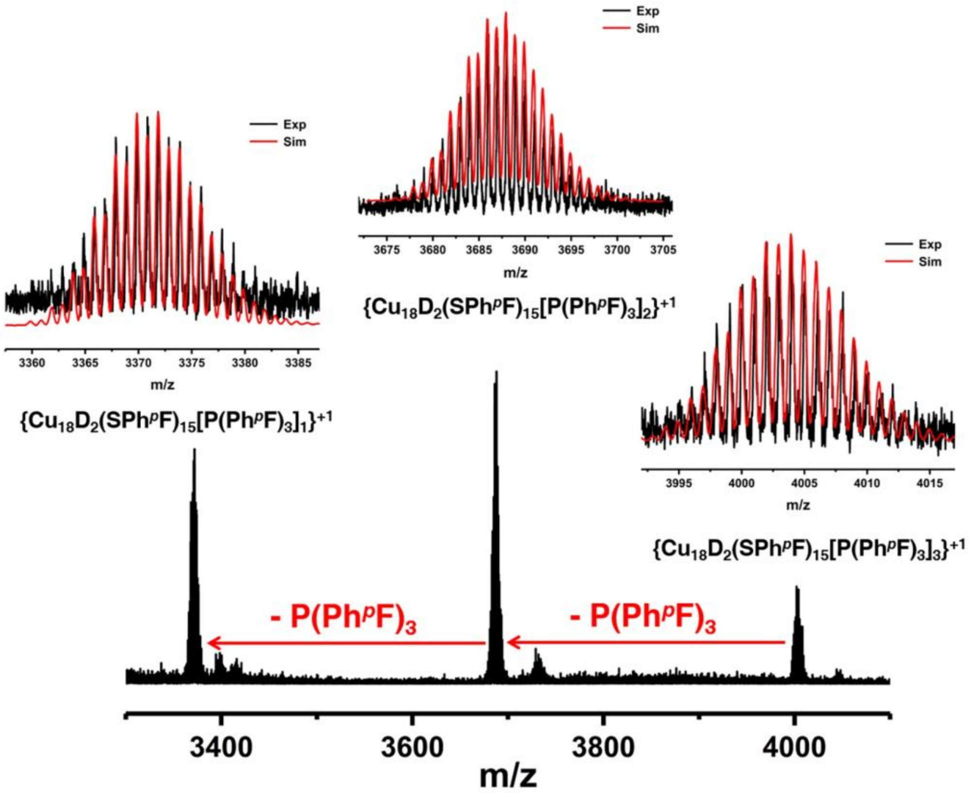


**Figure S3.** ESI-MS result of the **Cu18-D** nanocluster. Three mass signals were detected, including $\{Cu_{18}D_2(SPh^pF)_{15}[P(Ph^pF)_3]_1\}^{1+}$, $\{Cu_{18}D_2(SPh^pF)_{15}[P(Ph^pF)_3]_2\}^{1+}$, and $\{Cu_{18}D_2(SPh^pF)_{15}[P(Ph^pF)_3]_3\}^{1+}$. Insets: experimental (black lines) and calculated (red lines) isotope patterns.

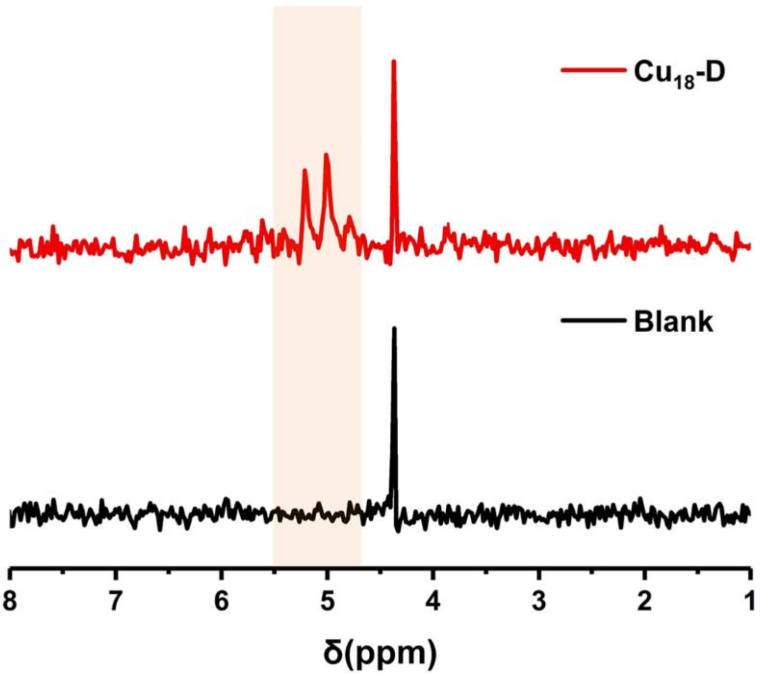


**Figure S4.** $^2$H NMR spectrum of **Cu18-D** (dissolved in $CH_2Cl_2$) showed two discrete signals at 5.00 and 5.11 ppm with a 1:1 area radio (red line).

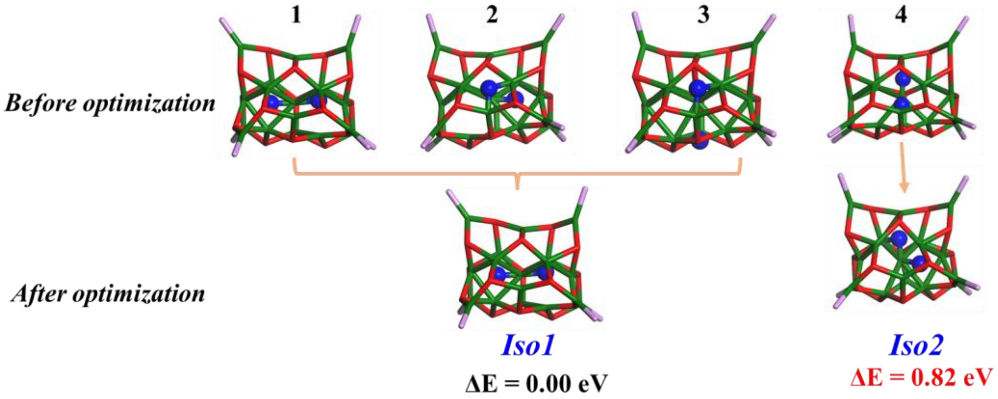


**Figure S5**. Schematic diagram of the four configurations of **$Cu_{18}$** clusters before and after DFT optimizations. Color labels: green, Cu; red, S; magenta, P; blue, H. For clarity, all C, F atoms and part of H atoms are omitted.

Considering that (i) the two $Cu_{11}$ subunits of the $Cu_{18}$ nanocluster were plane-symmetric along the $Cu_4(SPh^pF)_3$ face while some structural differences still existed and (ii) the two hydride signals located closely, we proposed that the two hydride ligands should be located into the cluster framework with two symmetric positions. As shown in Figure S5, four $Cu_{18}$ configurations with different hydride arrangements converged to two stable isomers (designated Iso1 and Iso2) after the geometry optimization, and the Iso1 exhibited a greater energetic stability compared to Iso2 by -0.82 eV. Additionally, two unstable $Cu_{18}$ configurations transformed into stable structure Iso1 after the geometry optimization, suggesting that the two hydride ligands were more likely to be symmetrically distributed at the hexahedral center of the $Cu_9$ kernel in the **$Cu_{18}$** cluster.

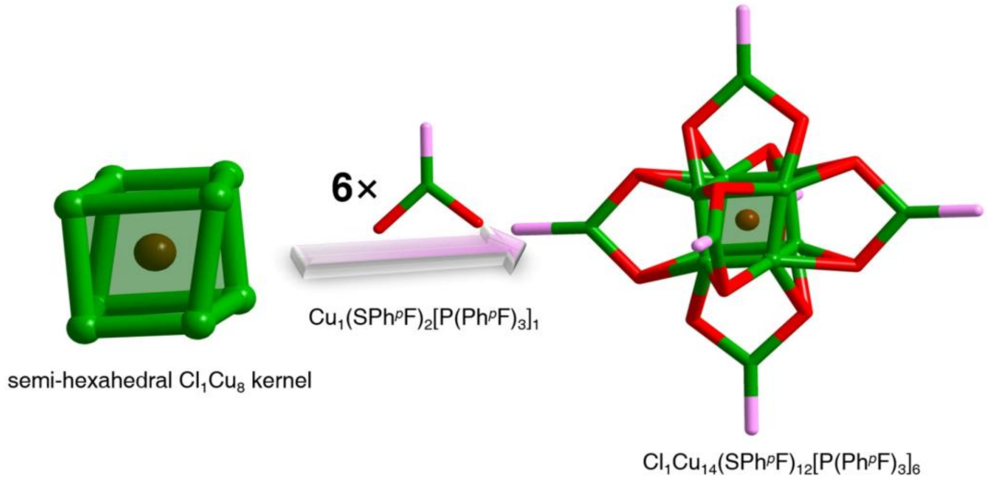


**Figure S6.** The overall structure of the **$Cu_{14}$** nanocluster contained a semi-hexahedral $Cl_1Cu_8$ kernel that was stabilized by six $Cu_1(SPh^pF)_2[P(Ph^pF)_3]_1$ motif-like structures from each face. Color labels: green, Cu; brown, Cl; magenta, P; red, S. For clarity, all C, F, and H atoms are omitted.

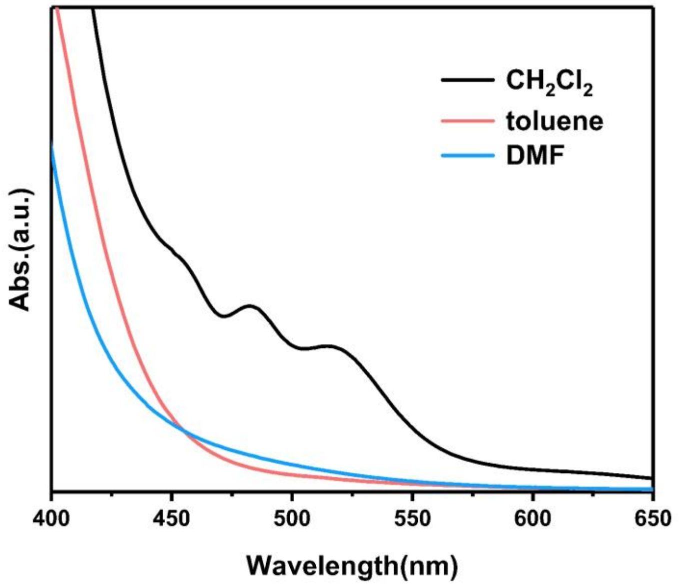


**Figure S7.** Optical absorptions of the photo-induced structural transformation of **Cu18** in different solvents. For the experiment, the concentration of the solution was 2.5 mg/mL, and the 365 nm irradiation time was 120 s.

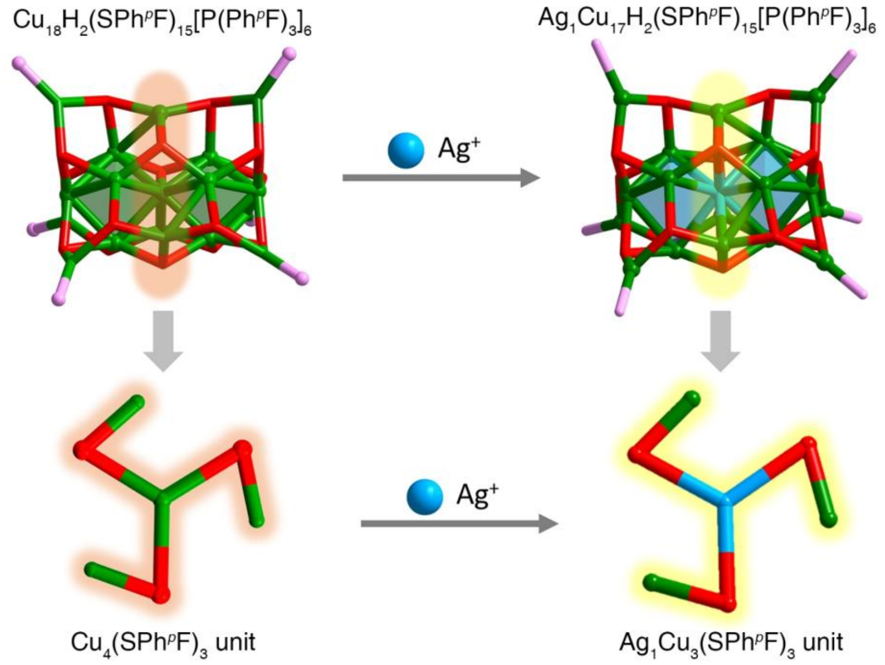


**Figure S8.** Total structures of **Cu18** and **Ag1Cu17** nanoclusters and the corresponding $Cu_4(SPh^pF)_3$ and $Ag_1Cu_3(SPh^pF)_3$ units, respectively. The introduced Ag heteroatom located at the innermost position of the cluster framework, and the shared $Cu_4(SPh^pF)_3$ face in **Cu18** was substituted by $Ag_1Cu_3(SPh^pF)_3$. Color labels: green, Cu; blue, Ag; magenta, P; red, S. For clarity, all C, F, and H atoms are omitted.

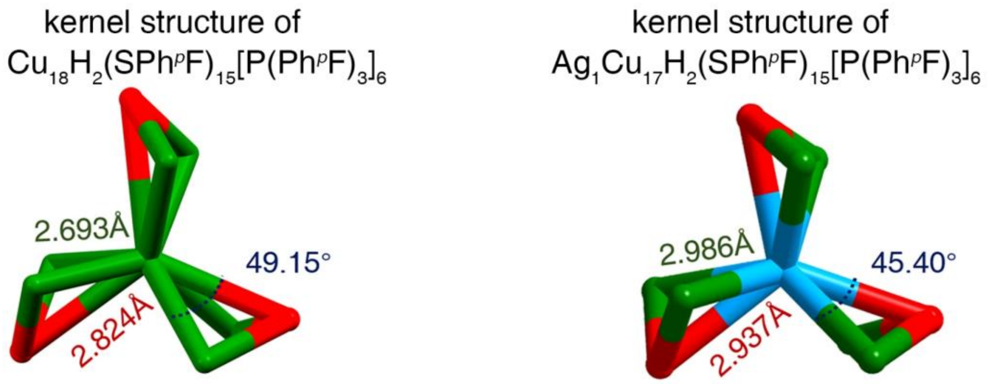


| | $Cu_{18}$ nanocluster | $Ag_1Cu_{17}$ nanocluster |
|---|---|---|
| M-Cu(Å) | 2.700 | 2.834 |
| M-S (Å) | 2.817 | 2.945 |
| ∠Cu-M-S (°) | 49.33 | 46.76 |

**Figure S9.** Comparison of the kernel structure of **$Cu_{18}$** and **$Ag_1Cu_{17}$** nanoclusters in terms of the interactions between the innermost Ag/Cu core and its adjacent Cu and S atoms. Color labels: green, Cu; blue, Ag; red, S.

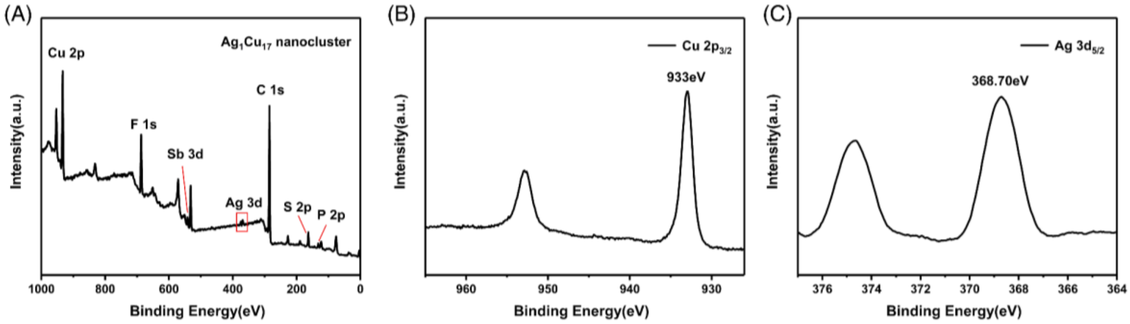


**Figure S10.** XPS results of (A) the **$Ag_1Cu_{17}$** nanocluster. (B) XPS results of the Cu binding energy in the **$Ag_1Cu_{17}$** nanocluster. (C) XPS results of the Ag binding energy in the **$Ag_1Cu_{17}$** nanocluster.

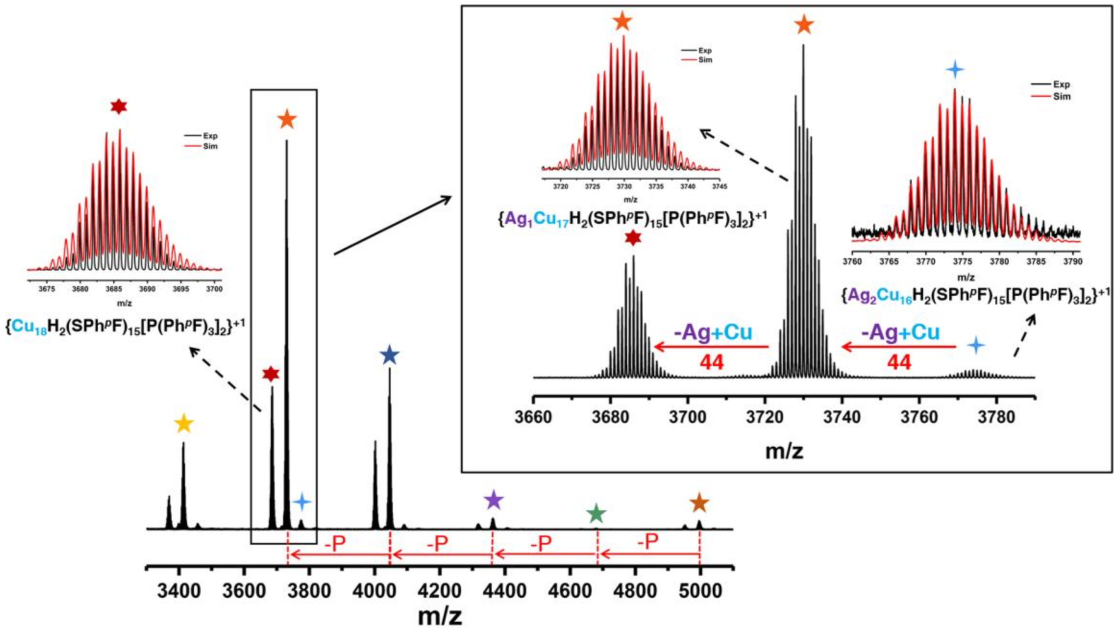


**Figure S11.** ESI-MS of the **$Ag_1Cu_{17}$** nanocluster. Several comparable mass signals of $Cu_{18}$, $Ag_1Cu_{17}$, and $Ag_2Cu_{16}$ nanoclusters were detected, demonstrating the flexibility of this cluster framework. Besides, the phosphine ligands in such nanoclusters followed an easy-to-dissociate state.

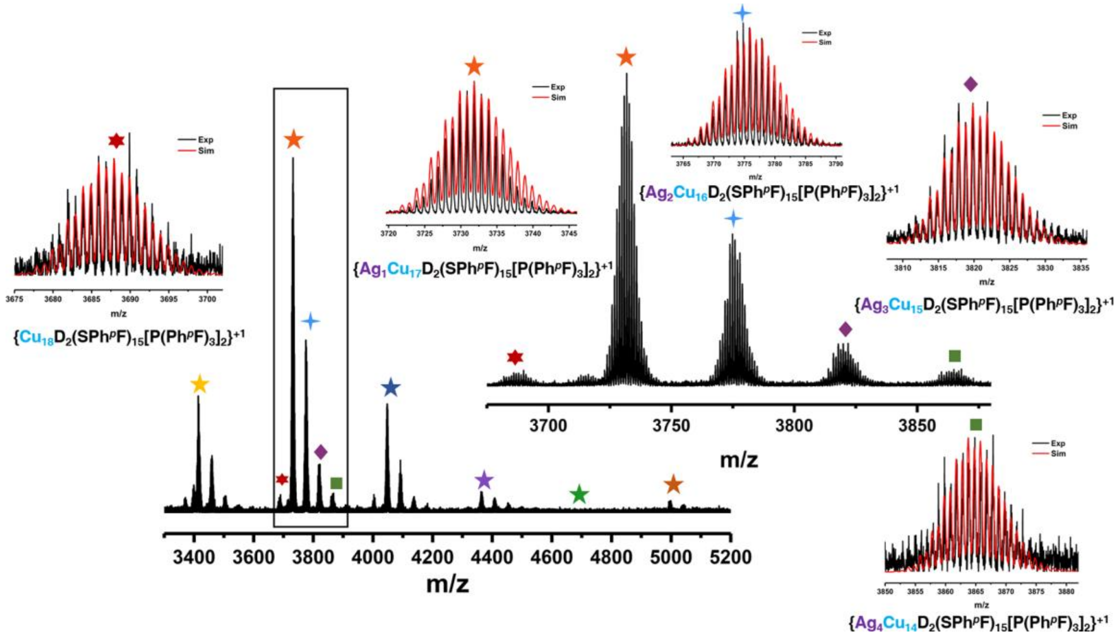


**Figure S12.** ESI-MS of the **$Ag_1Cu_{17}$** nanocluster. Several comparable mass signals of $Cu_{18}$, $Ag_1Cu_{17}$, $Ag_2Cu_{16}$, $Ag_3Cu_{15}$, and $Ag_4Cu_{14}$ nanoclusters were detected, demonstrating the flexibility of this cluster framework. Besides, the phosphine ligands in such nanoclusters followed an easy-to-dissociate state.

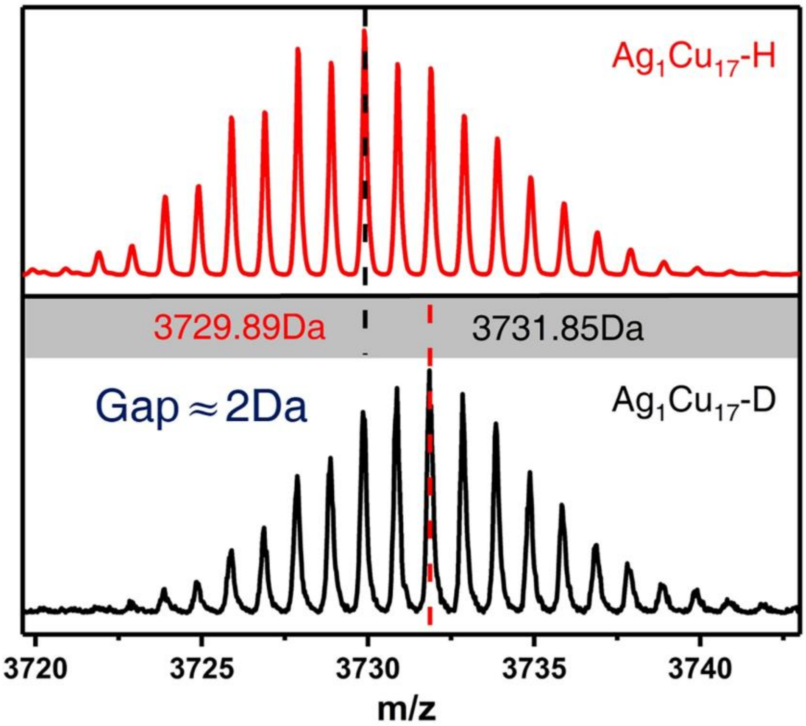


**Figure S13.** ESI-MS results of **$Ag_1Cu_{17}$-H** (red line) and **$Ag_1Cu_{17}$-D** (black line). The 2 Da mass gap indicated the presence of two hydride ligands in the cluster framework.

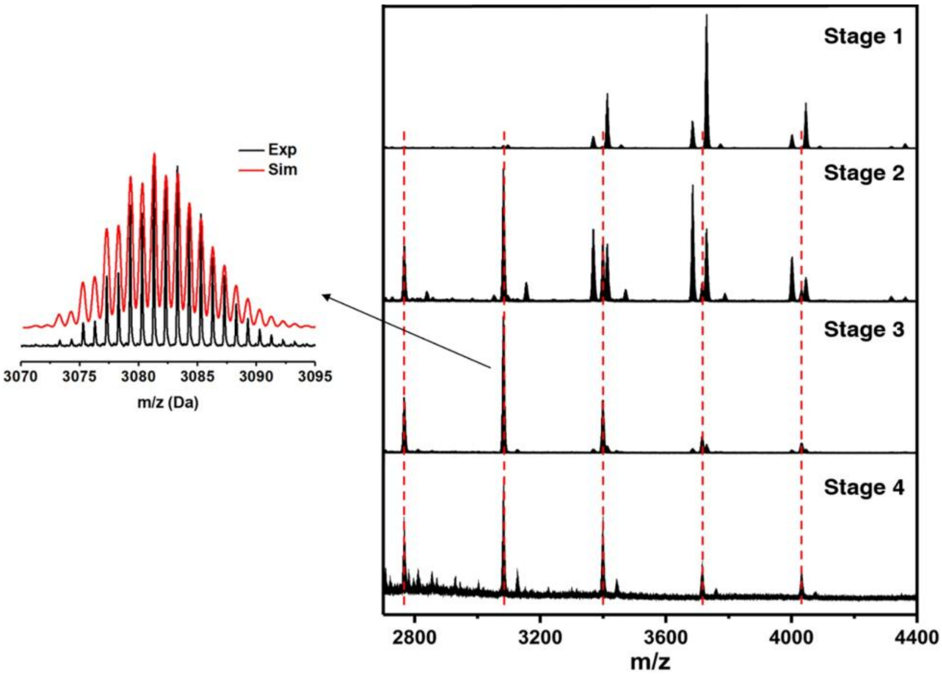


**Figure S14.** Time-dependent ESI-MS results of the photo-induced transformation from **$Ag_1Cu_{17}$** to **$Cu_{14}$**. (Left) experimental mass result of $\{Cl_1Cu_{14}(SPh^pF)_{12}[P(Ph^pF)_3]_2\}^{+1}$ (black line) and its calculated isotope pattern (red line)

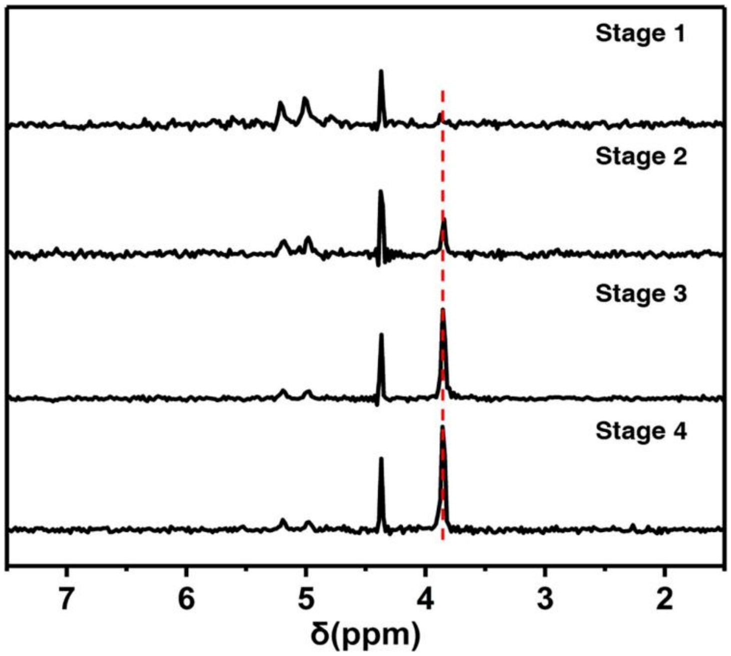


**Figure S15.** Time-dependent $^{2}$H NMR spectra of the photo-induced transformation from $\mathbf{Ag_1Cu_{17}}$ to $\mathbf{Cu_{14}}$ validated the gradual conversion from $\mathbf{Ag_1Cu_{17}}$ to $\mathbf{Cu_{14}}$.

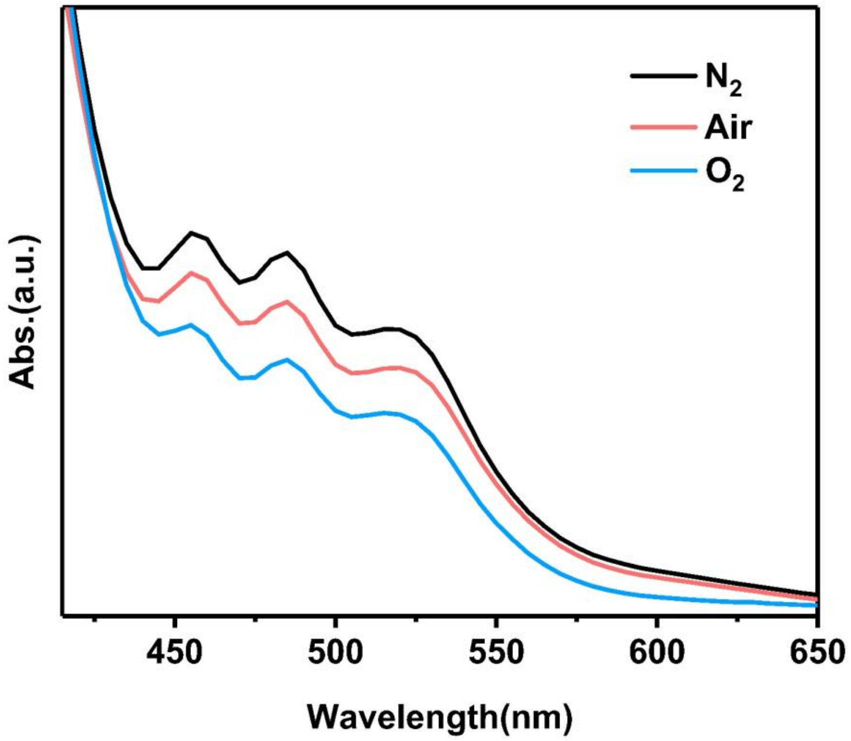


**Figure S16.** Optical absorptions of the photo-induced structural transformation of $\mathbf{Ag_1Cu_{17}}$ in $CH_2Cl_2$ under different gas atmospheres. For the experiment, the concentration of $\mathbf{Ag_1Cu_{17}}$ was 2.5 mg/mL, and the 365 nm irradiation time was 120 s.

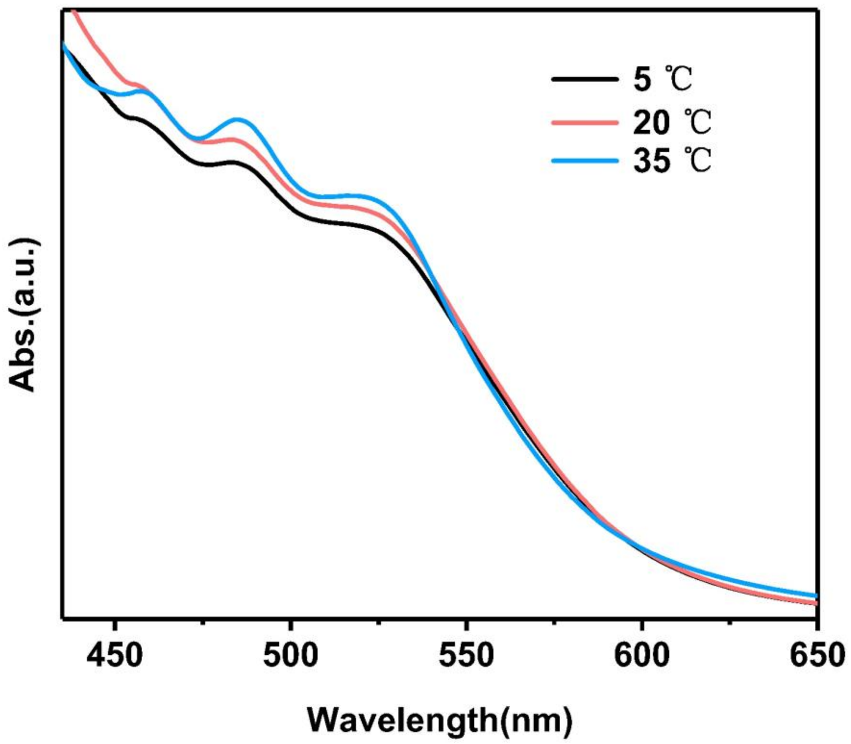


**Figure S17.** Optical absorptions of the photo-induced structural transformation of **$Ag_1Cu_{17}$** in $CH_2Cl_2$ under different temperatures. For the experiment, the concentration of **$Ag_1Cu_{17}$** was 2.5 mg/mL, and the 365 nm irradiation time was 120 s.

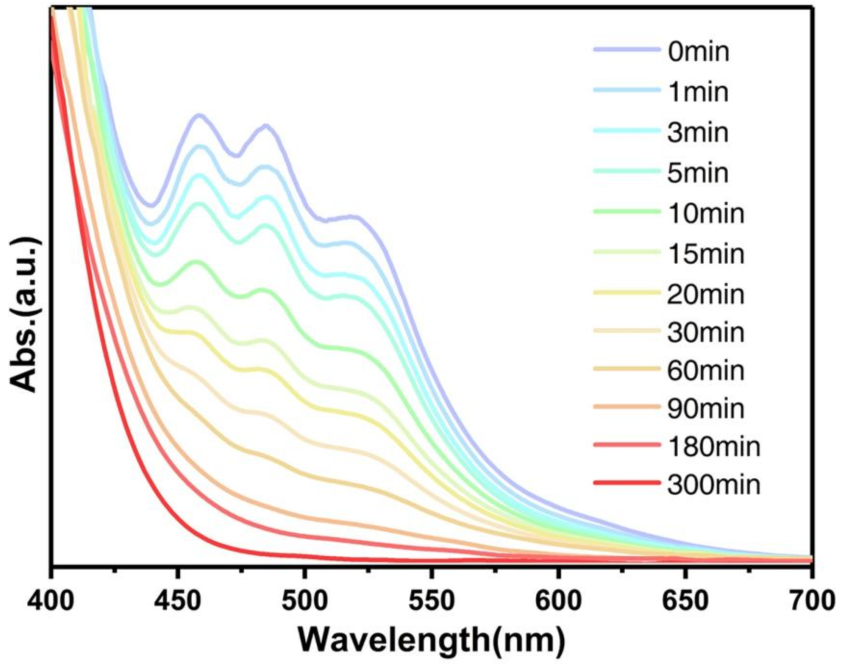


**Figure S18.** Time-dependent optical absorption results of the $Cu_{14}$ nanocluster (dissolved in $CH_2Cl_2$) under the 365 photoexcitation suggested its metastable characterization under such a condition.

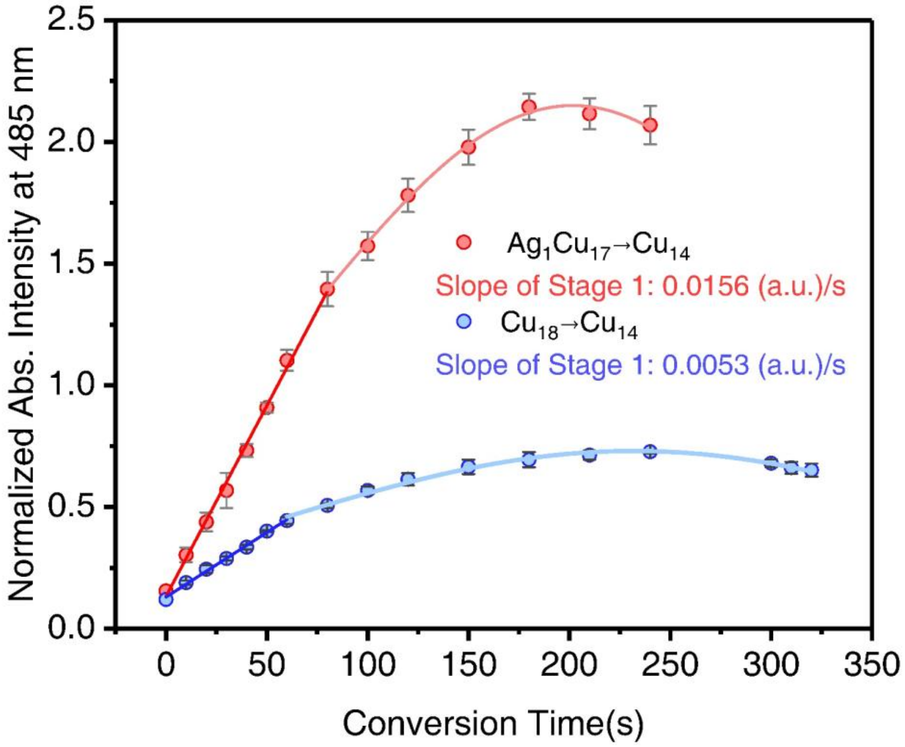


**Figure S19.** Time-dependent concentration of the photoinduced generated $\mathbf{Cu_{14}}$ nanoclusters from $\mathbf{Cu_{18}}$ or $\mathbf{Ag_1Cu_{17}}$. Data are presented as mean ± standard deviation (SD) from n = 3 independent experiments. For the experiment, the initial concentration of $\mathbf{Cu_{18}}$ and $\mathbf{Ag_1Cu_{17}}$ was 2.5 mg/mL.

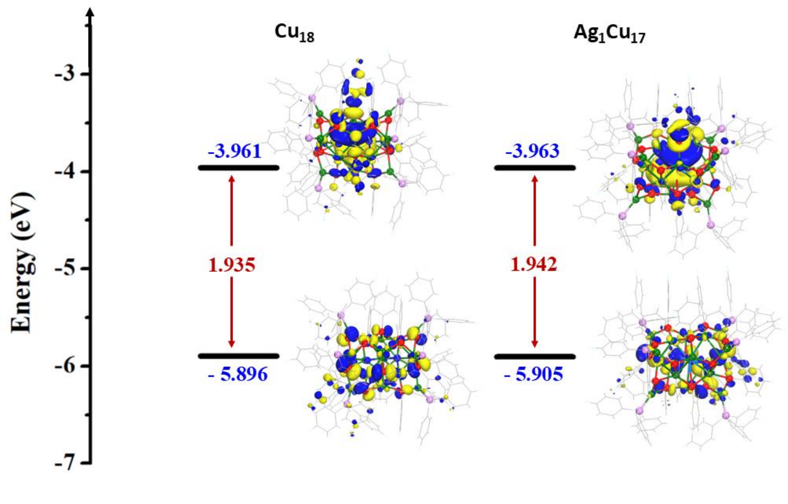


**Figure S20.** Contour plots and energy levels of $\mathbf{Cu_{18}}$ and $\mathbf{Ag_1Cu_{17}}$ clusters. All the isovalues for the molecular orbital plots are set to 0.02.

As shown in Figure S16, the HOMO and LUMO energy levels and the HOMO-LUMO gap were the same for $\mathbf{Cu_{18}}$ and $\mathbf{Ag_1Cu_{17}}$ clusters. In this context, the Ag doping would not significantly change the electronic structure of $\mathbf{Cu_{18}}$, suggesting that the high conversion efficiency from $\mathbf{Ag_1Cu_{17}}$ to $\mathbf{Cu_{14}}$ was

not related to this factor. The transition from **$Cu_{18}$** or **$Ag_1Cu_{17}$** to **$Cu_{14}$** occurred under photoinduced conditions, indicating that the conversion rate was affected by both electronic structures and geometrical configurations of clusters in the excited-state. Subsequently, we examined the stability of the excited-state structures of **$Ag_1Cu_{17}$** and **$Cu_{18}$** clusters.

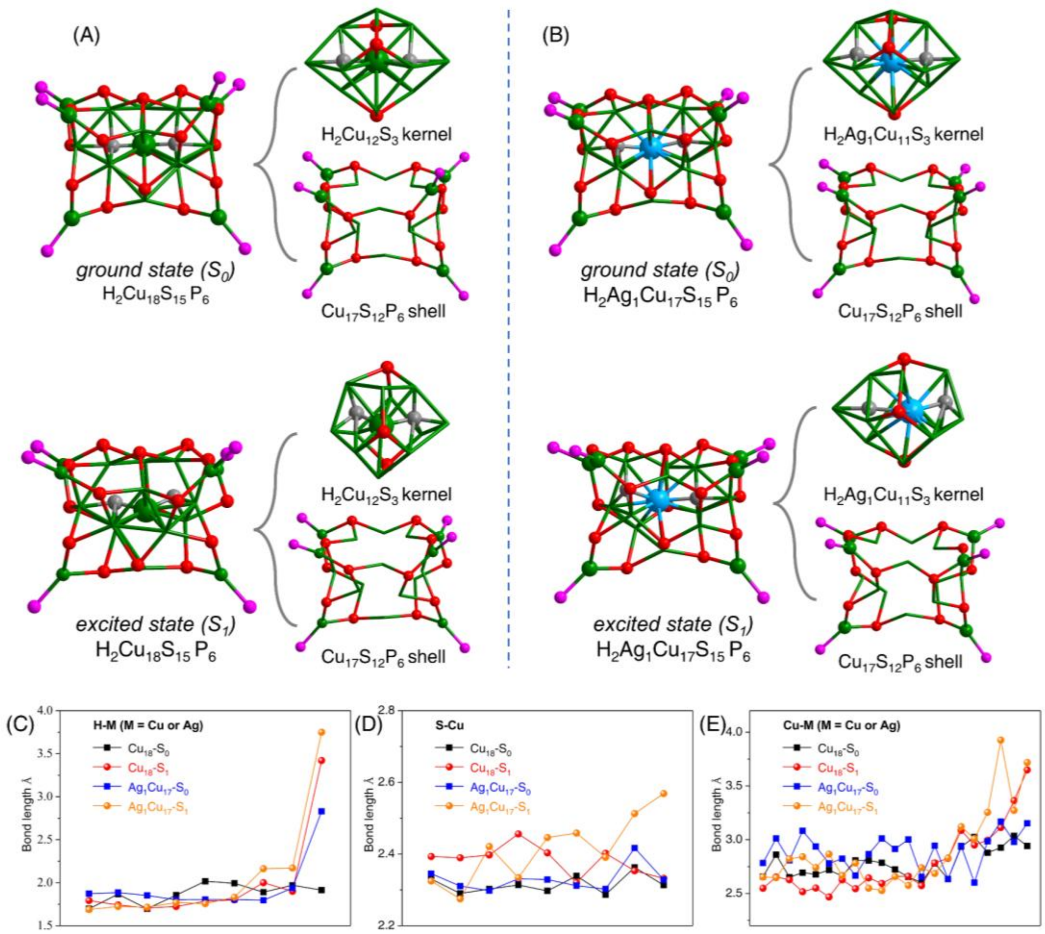


**Figure S21.** Calculation results of the photoinduced conversion from **$Cu_{18}$** or **$Ag_1Cu_{17}$** to **$Cu_{14}$**. Optimization ground and excited state structures of (A) **$Cu_{18}$** and (B) **$Ag_1Cu_{17}$** nanoclusters. Color labels: green, Cu; blue, Ag; magenta, P; red, S; brown, Cl; grey, H. For clarity, all of C, F atoms, and some of H atoms are omitted. (C) H-M (M = Cu or Ag) bond lengths, (D) S-Cu bond lengths, and (E) Cu-M (M = Cu or Ag) bond lengths in the internal $H_2M_1Cu_{11}S_3$ core of $S_0$ and $S_1$ structures of **$Cu_{18}$** and **$Ag_1Cu_{17}$** nanoclusters.

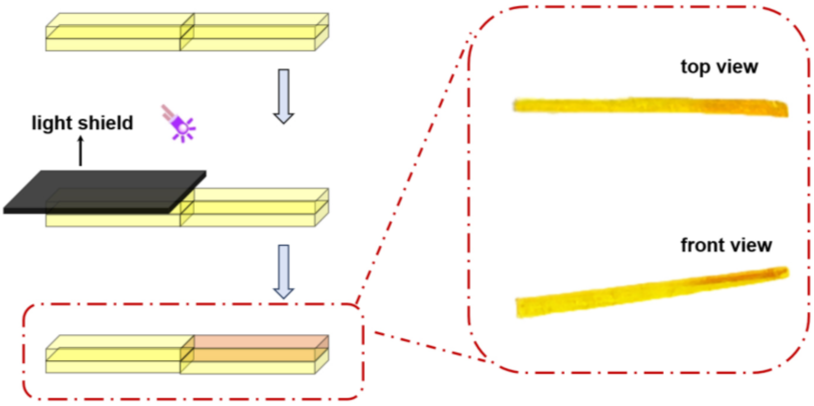


**Figure S22.** Schematic diagram and digital photos (insets) of the sole $\mathbf{Ag_1Cu_{17}}$ crystal photoinduced transformation, and the lightproof area on the left half of the crystal.

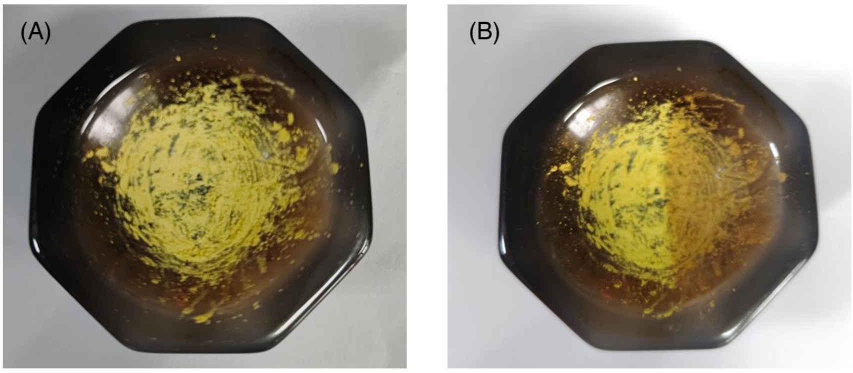


**Figure S23.** Digital photo of light-induced transition from $\mathbf{Ag_1Cu_{17}}$ to $\mathbf{Cu_{14}}$ in powder state, and the lightproof area on the left. (A) Before photoexcitation. (B) After photoexcitation.

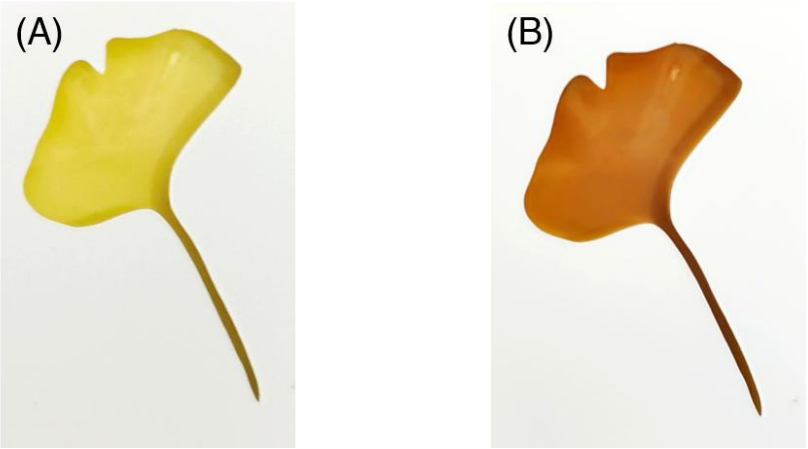


**Figure S24.** Digital photo of light-induced transition from $\mathbf{Ag_1Cu_{17}}$ to $\mathbf{Cu_{14}}$ in thin films. (A) Before photoexcitation. (B) After photoexcitation.

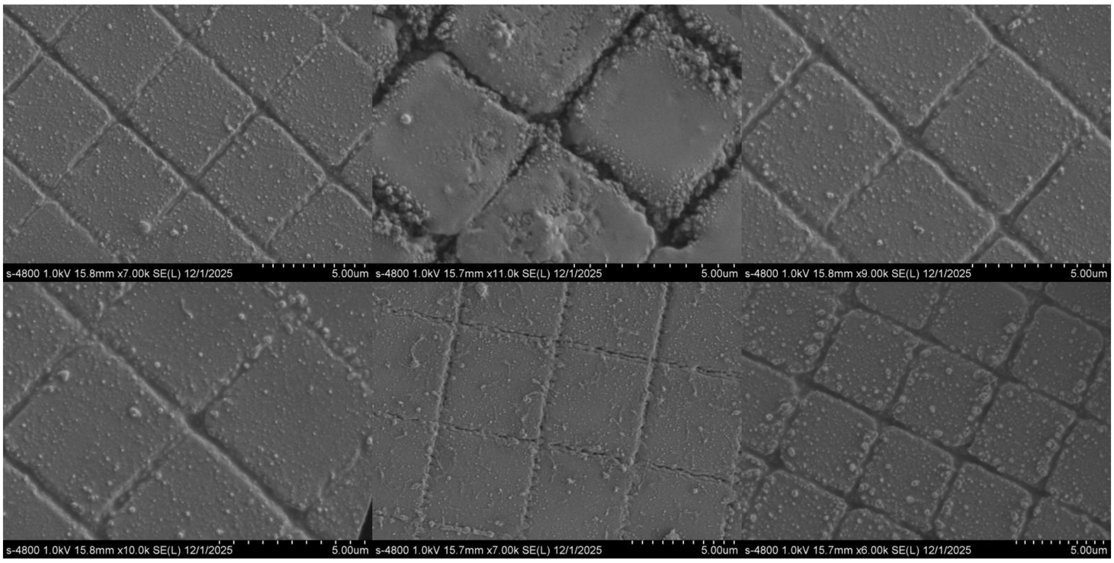


**Figure S25.** SEM images of the geometric patterns on the $\mathbf{Ag_1Cu_{17}}$ crystal.

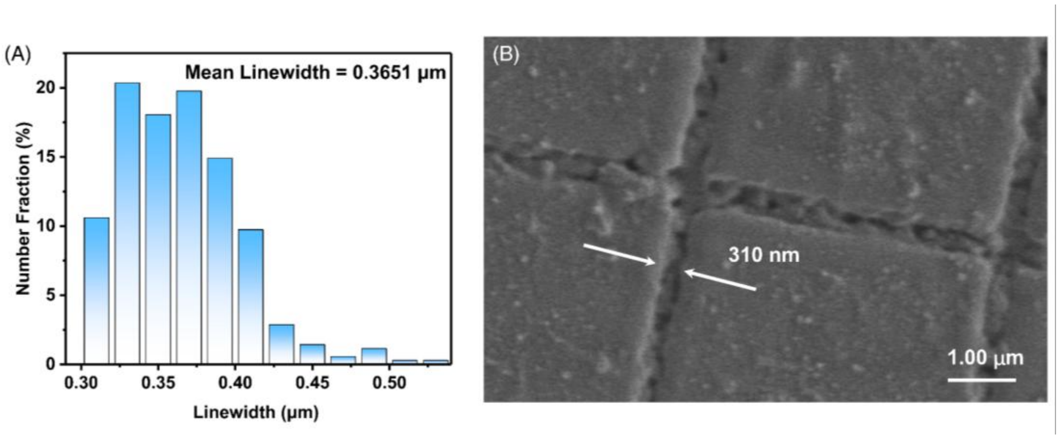


**Figure S26.** (A) Size distribution histogram of written lines. Statistical data are derived from six identical patterns, yielding an average line width of 0.3651 μm (CV = 4.6%). (B) SEM images of the minimum written lines.

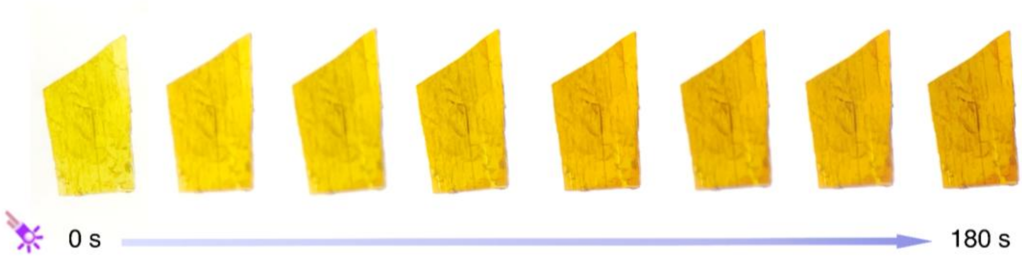


**Figure S27.** Time-dependent photographs of photoinduced color evolution in $\mathbf{Ag_1Cu_{17}}$ crystal upon 365 nm irradiation.

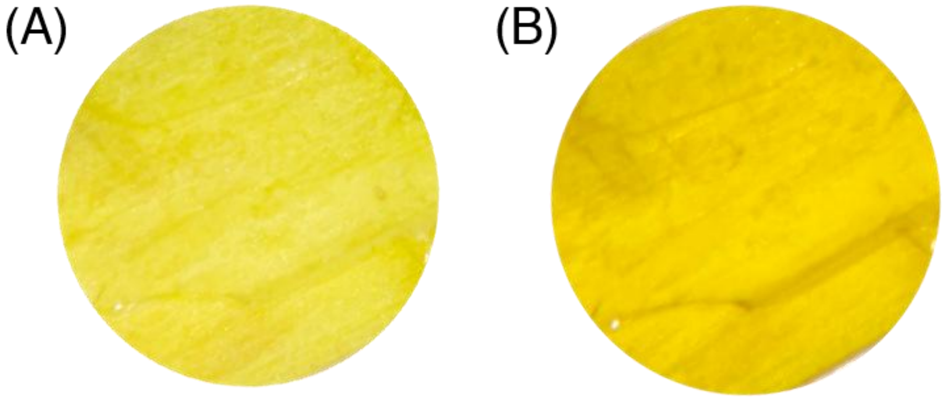


**Figure S28.** Digital photo of a single **$Cu_{18}$** crystal photoinduced transformation. (A) Before photoexcitation. (B) After photoexcitation.

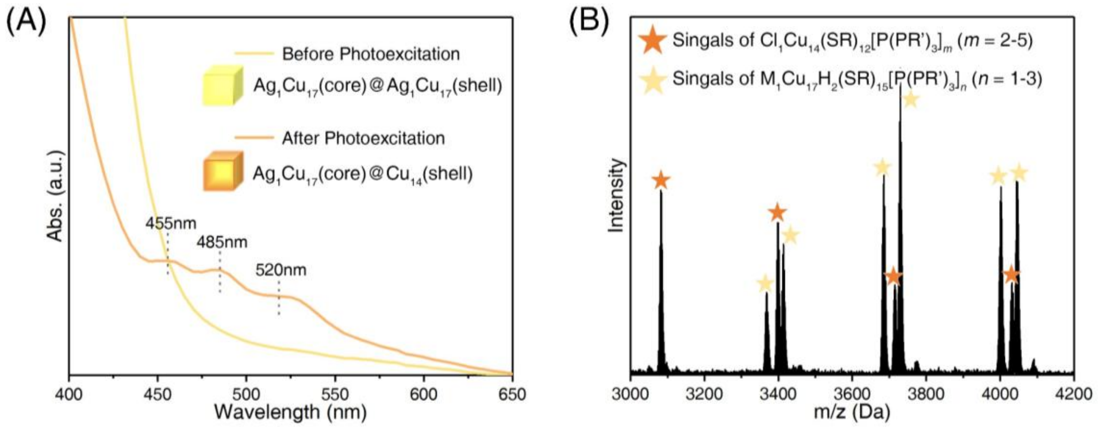


**Figure S29.** (A) Optical absorptions of cluster crystals before and after the 365 nm photoexcitation. (B) ESI-MS results of cluster crystals before and after the 365 nm photoexcitation.

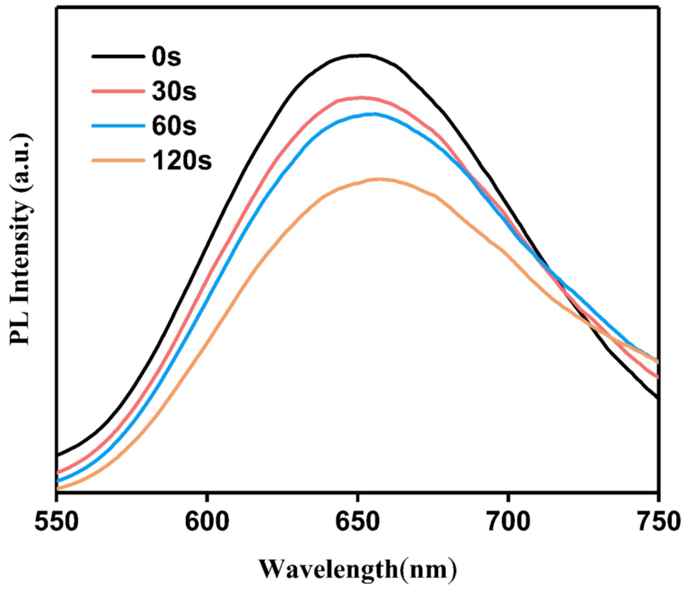


**Figure S30.** Time-dependent fluorescence spectra of the photoinduced conversion for the **$Ag_1Cu_{17}$** crystal ($\lambda_{ex}$ = 445nm). The fluorescence intensity at 653 nm gradually decreased as the duration of light radiation increased.

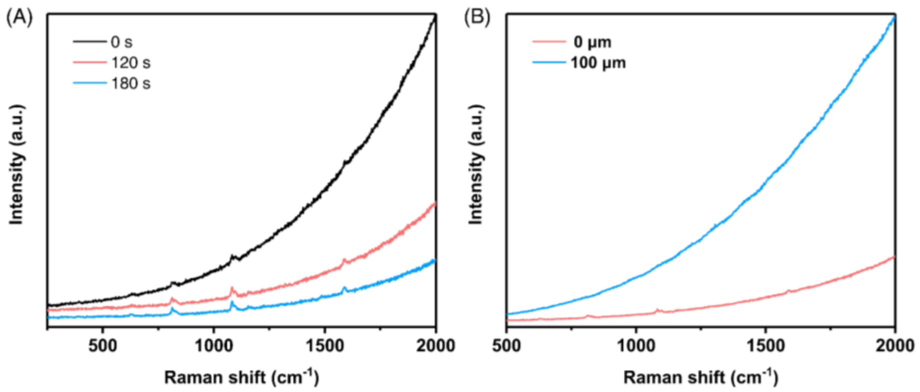


**Figure S31.** (A) Time-dependent Raman spectra of the photoinduced conversion for the **$Ag_1Cu_{17}$** crystal. (B) Micro-scale Raman spectra of **$Ag_1Cu_{17}$** (core)@**$Cu_{14}$** (shell) configuration.

Raman signals can be significantly affected by fluorescence background interference, with the extent of baseline elevation being proportional to the fluorescence intensity. Comparison of the Raman spectra before and after illumination revealed no shift in the positions of the characteristic peaks; however, a marked difference in the fluorescence background was observed in Figure S31A. Micro-scale Raman spectroscopy acquired at the outer layer (surface, 0 μm) exhibited a weak fluorescence background, consistent with the presence of a **$Cu_{14}$** shell. In contrast, spectra from the interior region (depth of 100 μm) displayed a significantly stronger fluorescence signal, characteristic of the unchanged **$Ag_1Cu_{17}$** core. These findings collectively provide evidence to support the formation of the heterogeneous **$Ag_1Cu_{17}$** (core)@**$Cu_{14}$** (shell) configuration.

## 4. Crystal Data and Structure Refinement

**Table S1.** Crystal data and structure refinement for **$Cu_{18}$** nanocluster.

| | |
|---|---|
| Empirical formula | C198H132Cu18F39P6S15Sb1 |
| Formula weight | 5184.22 |
| Temperature/K | 120 |
| Crystal system | orthorhombic |
| Space group | Pbca |
| a, b, c/Å | 36.8203 (4), 32.2641 (6), 37.2014 (5) |
| A, β, γ/° | 90, 90, 90 |
| Volume/Å$^3$ | 44194.3 (9) |
| Z | 8 |
| $\rho_{calc}$ g/cm$^3$ | 1.558 |
| μ/mm$^{-1}$ | 5.179 |
| F(000) | 20592 |
| Crystal size/mm$^3$ | 0.2 × 0.15 × 0.15 |
| Radiation | Cu Kα (λ = 1.54186) |
| 2Θ range for data collection/° | 11.906 to 124.994 |
| Index ranges | $-41 \le h \le 43$, $-37 \le k \le 24$, $-43 \le l \le 34$ |
| Reflections collected | 213810 |
| Independent reflections | 36510 [$R_{int}$ = 0.1339, $R_{sigma}$ = 0.1336] |
| Data/restraints/parameters | 36510/2814/2497 |
| Goodness-of-fit on $F_2$ | 0.888 |
| Final R indexes [I>=2σ (I)] | R1 = 0.0832, wR2 = 0.2117 |
| Final R indexes [all data] | R1 = 0.1346, wR2 = 0.2357 |
| Largest diff. peak/hole / e Å$^{-3}$ | 2.11/-2.74 |

**Table S2.** Crystal data and structure refinement for **$Ag_1Cu_{17}$** nanocluster.

| | |
|---|---|
| Empirical formula | C198H134Ag1Cu17F39P6S15Sb1 |
| Formula weight | 5230.56 |
| Temperature/K | 120 |
| Crystal system | monoclinic |
| Space group | I2/a |
| a, b, c/Å | 32.2725 (1), 36.3369 (1), 36.3503 (1) |
| A, β, γ/° | 90, 94.148 (3), 90 |
| Volume/$Å^3$ | 42516 (3) |
| Z | 8 |
| $\rho_{calc}$ g/$cm^3$ | 1.634 |
| μ/$mm^{-1}$ | 5.999 |
| F(000) | 20752 |
| Crystal size/$mm^3$ | 0.18 × 0.147 × 0.11 |
| Radiation | Cu Kα (λ = 1.54186) |
| 2Θ range for data collection/° | 6.34 to 132.54 |
| Index ranges | -33 ≤ h ≤ 37, -41 ≤ k ≤ 34, -41 ≤ l ≤ 29 |
| Reflections collected | 97601 |
| Independent reflections | 33536 [$R_{int}$ = 0.0814, $R_{sigma}$ = 0.0944] |
| Data/restraints/parameters | 33536/2802/2310 |
| Goodness-of-fit on $F_2$ | 1.257 |
| Final R indexes [I>=2σ (I)] | R1 = 0.1197, wR2 = 0.3211 |
| Final R indexes [all data] | R1 = 0.1921, wR2 = 0.3880 |
| Largest diff. peak/hole / e $Å^{-3}$ | 3.181/-1.946 |
